# Hierarchical Modular Architecture Enabling Intelligent Dynamic Thermal Management and Superior Electromagnetic Interference Shielding

**DOI:** 10.1007/s40820-026-02140-9

**Published:** 2026-03-18

**Authors:** Qi-Fan Xuan, Pei-Yan Zhao, Hualong Peng, Shan Zhang, Bo Cai, Fang-Yu Niu, Martin C. Koo, Xiao-Bo Sun, Xiangyu Jiang, Guang-Sheng Wang

**Affiliations:** 1 State Key Laboratory of Bioinspired Interfacial Materials Science, Bioinspired Science Innovation Center, Hangzhou International Innovation Institute, Beihang University, Hangzhou, 311115 People’s Republic of China; 2 School of Chemistry, Beihang University, Beijing, 100191 People’s Republic of China

**Keywords:** MXene, Hierarchical modular design, Thermal management, Temperature–humidity sensing, Electromagnetic interference shielding

## Abstract

Integrated wearable thermal management technologies have greatly enhanced human adaptability to complex environments. However, conventional thermal management strategies, which lack environmental risk perception and stable human–machine interaction, are increasingly inadequate for ensuring personal health. Here, we introduce a hierarchical modular design strategy to develop a wearable intelligent thermal management film with robust electromagnetic interference (EMI) shielding capabilities. A sensitive biomimetic serpentine dual-mode temperature–humidity sensing module is coupled with a low-power electro-/photothermal conversion module to enable intelligent thermal regulation. The resulting thermal management system offers stable and sensitive front-end temperature–humidity monitoring, alongside low-power electrothermal (51.79 °C at 1.5 V) and photothermal (56.38 °C at 45.51 mW cm^−2^) temperature regulation capabilities. Additionally, the system exhibits outstanding EMI shielding performance, with an EMI SE/t value of 1600 dB mm^–1^ at a thickness of just 35 μm, ensuring stable signal transmission. The hierarchical modular design enables functional allocation with higher, thereby optimizing material performance while enhancing the decoupling and synergistic effects between different functionalities. These findings provide a scalable and practical pathway for the multifunctional integration and performance optimization of next-generation flexible wearable electronic composites.

**Supplementary Information:**

The online version contains supplementary material available at 10.1007/s40820-026-02140-9.

## Highlights

- Hierarchical modular architecture for integrated design and functional optimization.
- Development of an intelligent monitoring and feedback-driven thermal management system, providing not only stable and sensitive front-end temperature and humidity monitoring, but also low-power thermoelectric (1.5 V, 51.79 °C) and photothermal (45.51 mW cm^-2^, 56.38 °C) dual complementary temperature control capabilities for the back end.
- Multiscale and scalable conductive gradient for exceptional electromagnetic interference shielding performance (35 μm, 1600 dB mm^-1^), ensuring stable signal transmission across the system.

**Supplementary Information:**

The online version contains supplementary material available at 10.1007/s40820-026-02140-9.

## Graphical abstract

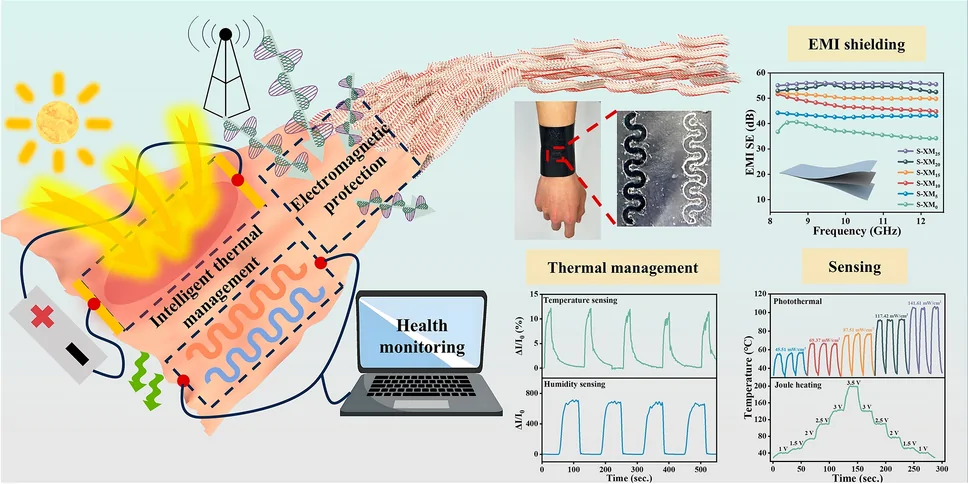

## Introduction

Maintaining thermal stability of the human body is essential for personal comfort and health [1, 2]. Given the increasing frequency of extreme weather events and unpredictable temperature changes, smart wearable thermal management devices offer significant advantages over traditional insulation strategies [3]. These intelligent devices provide efficient and controllable heating while facilitating early detection of potential health risks, enhancing the response ability to respond to complex environmental threats [4]. However, electromagnetic interference (EMI) generated by the electronic components within these devices poses a serious threat to their reliable operation and presents potential risks to human health [5–7]. To address these challenges, there is an urgent need for protective materials specifically designed for flexible wearable devices, which integrates both electromagnetic shielding and intelligent thermal regulation functions [8].

The integration of EMI shielding and thermal regulation functions in flexible wearable film platforms primarily relies on the incorporation of materials that exhibit high electrical conductivity, outstanding electrothermal and photothermal properties. Sensitive materials such as carbon nanotubes [9, 10], silver nanowires [11], liquid metals [12, 13], MXenes [14, 15], and graphene [16, 17], have played a pivotal role in imparting multifunctionality to flexible wearable films. Materials incorporating flexible polymer substrates have been successfully integrated using techniques such as vacuum filtration [18], spray coating [19, 20], and blade coating [21] to fabricate films that meet the requirements for both EMI shielding and personal thermal regulation. However, an independent electrothermal and photothermal heating module alone is insufficient to achieve real-time temperature monitoring and adaptive thermal feedback. To realize intelligent thermal regulation in wearable applications, the urgent need for the integration of sensing technologies arises [4]. Among various sensing approaches, temperature and humidity sensing has emerged as a critical focal point for research. Continuous, real-time monitoring of skin temperature and humidity is essential for responding promptly to environmental threats to human health [22, 23]. Yet, when integrating sensors into wearable systems, considerations of sensor structure design, user comfort, and system-level optimization are crucial to ensuring reliable signal fidelity and multifunctional coupling protection [24].

In this study, we propose a hierarchical modular design strategy for the fabrication of a multifunctional-layered film system based on carboxylated styrene butadiene rubber (XSBR) and MXene. The resulting XSBR/MXene (XM) film system integrates efficient EMI shielding, back-end electro-/photothermal conversion, and front-end temperature–humidity sensing functionalities. This film demonstrates exceptional EMI shielding performance (EMI SE/t up to 1600 dB mm^−1^ at 35 μm), providing a solid foundation for personal electromagnetic protection and reliable signal transmission. Additionally, the film exhibits stable, controllable, low-power Joule heating (51.79 °C at 1.5 V), and photothermal properties (56.38 °C at 45.51 mW cm^−2^), enabling a reliable collaborative heating mechanism that supports back-end thermal regulation in personal thermal management applications. The integration of temperature and humidity sensors further extends the front-end real-time biosignal monitoring capabilities of the system, enabling intelligent personal thermal management when combined with back-end thermal regulation. The hierarchical modular design strategy enables more precise functional allocation and material performance optimization, thus enhancing the decoupling and synergistic effects between different functions while improving the scalability of the structure. This approach provides a simple yet reliable fabrication method for multifunctional integration and synergistic optimization of composite materials in next-generation flexible wearable electronics.

## Experimental Section

### Materials

Ti_3_AlC_2_ powders (400 mesh) were purchased from Jilin 11 Technology Co., Ltd. Carboxylic styrene butadiene rubber (XSBR, YF-0.19, solid content 49.69%) latex was produced by Wuhan Fengyao-Tonghui Chemical Products Co., Ltd. Lithium fluoride (LiF, ≥ 99.99%), dicumyl peroxide (DCP), and potassium hydroxide (KOH, 99%) were provided by Shanghai McLean Biochemical Technology Co., Ltd. Polyvinyl alcohol (PVA, alcohol solubility: 87.0–89.0 mol%, viscosity: 80.0–110.0 mPa s) was purchased from Shanghai Aladdin Biochemical Technology Co., Ltd. poly(3,4-ethylene dioxythiophene): poly(styrenesulfonate) (PEDOT:PSS 1000), purchased from Heraeus, German. Hydrochloric acid (HCl, 36% ~ 38%) was obtained from Beijing Modern Oriental Technology Development Co., Ltd. All reagents were of analytical grade and used without further purification.

### Preparation of XSBR/MXene Film

0.2 g of DCP vulcanizing agent was added to 20 g of XSBR latex, and the mixture was stirred at 50 °C and 650 rpm for 30 min to obtain XSBR latex with a solid content of 50.19%. The MXene concentrated suspension (30 mg mL^−1^) was then added to the XSBR latex at different weight ratios (0, 5, 10, 15, 20, and 25 wt%). The resulting XM latex mixtures, with varying MXene contents, were applied to a smooth polypropylene substrate using the blade coating method (MSK-AFA-L1000) with a 20-cm blade length, 0.15 mm blade height, and a coating speed of 3 cm s^−1^, while maintaining a temperature of 25 ± 3 °C and a relative humidity of 40 ± 5% during the coating process. The films were vacuum-dried at 40 °C to form single-layer XM films.

For the second layer, an XM latex mixture containing 50 wt% MXene was applied with a 0.20-mm blade height and the same coating speed. After vacuum-drying at 40 °C, a double-layer XM film was obtained. Additional layers with different MXene contents (0, 5, 10, 15, 20, and 25 wt%) were applied using the same blade method, adjusting the blade height to 0.30 mm. The multilayer film was dried and easily peeled from the substrate. Finally, the film was hot-pressed at 160 °C for 10 min to form the sandwich-structured XM (S-XM) film. The S-XM films, with varying MXene contents in the outer layers, were labeled as S-XM_0_, S-XM_5_, S-XM_10_, S-XM_15_, S-XM_20_, and S-XM_25_.

### S-XM Film Surface Temperature/Humidity Sensor Assembly

The XSBR insulating layer was first blade coating onto the outer surface of the S-XM_20_ film. A polyimide (PI) tape was adhered to a glass slide and laser-patterned according to the predefined design with a KRDB-CO_2_/30 W laser marking system (Coretronic Laser Intelligent Manufacturing Co., Ltd.), generating a skeletonized PI stencil. This stencil was gently peeled from the glass substrate and laminated onto the XSBR-coated S-XM_20_ film. PEDOT:PSS and PVA/KOH precursor solutions were then successively blade coating through the stencil and dried. Finally, the PI tape was removed, leaving well-defined conductive and electrolyte patterns on the film surface, thus completing sensor fabrication.

## Results and Discussion

### Preparation and Characterization of Layered XM Films

**Fig. 1:**
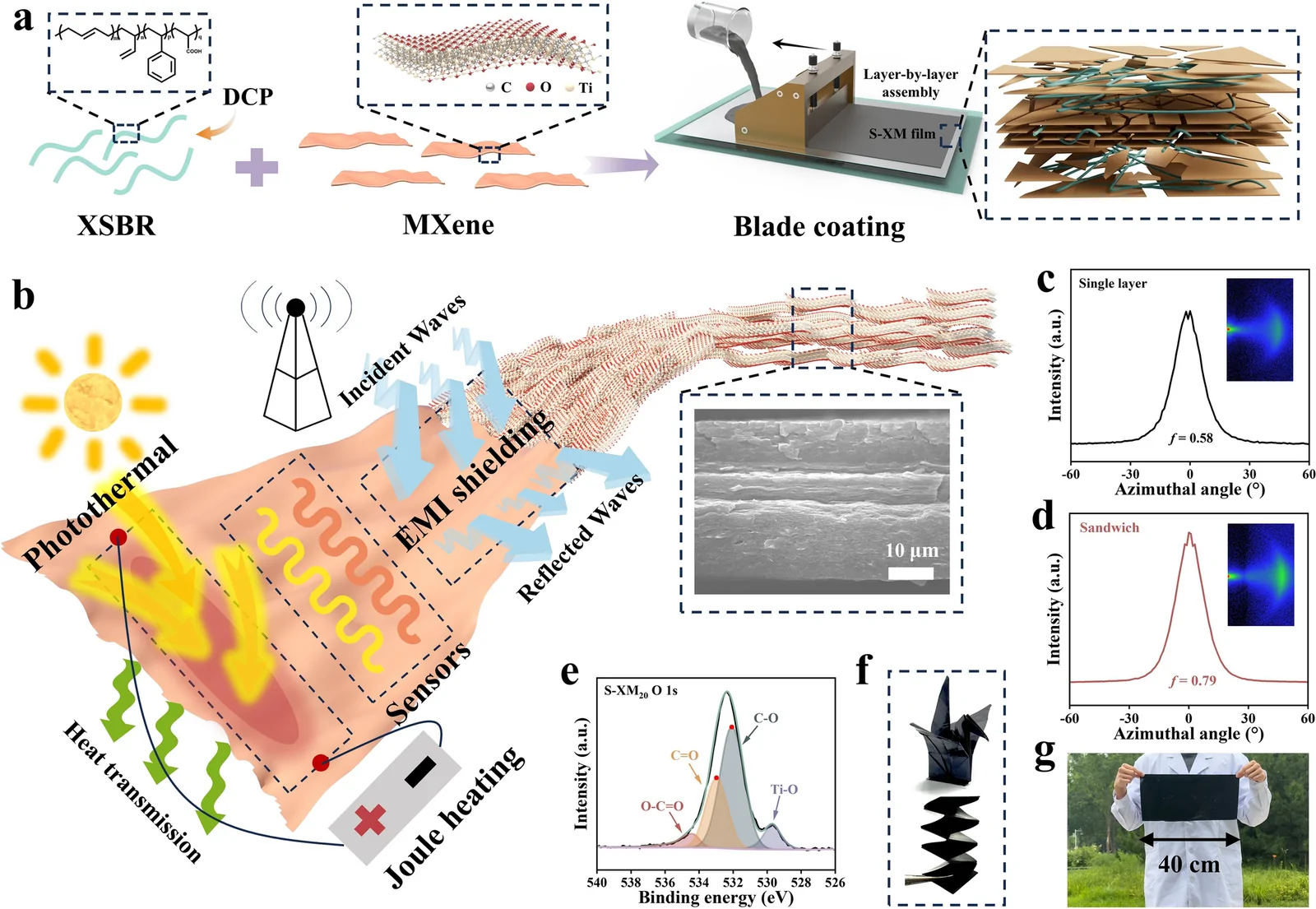
Assembly route and characterization scheme of multifunctional composite films. **a** Process for preparing S-XM film framework using a layer-by-layer blade coating method. **b** Schematic diagram of the film integrating EMI shielding, personal thermal management, and temperature–humidity signal monitoring functions, along with the cross-sectional SEM image of the S-XM film framework. WAXS pattern for an incident Cu-Ka X-ray beam parallel to the film plane. The azimuthal scan profiles were recorded for the (002) peak of **c** single-layer XM film and **d** S-XM film. **e** O 1*s* XPS spectra of S-XM films. **f** Photographs of S-XM films flexibly folded into “paper cranes” and “spring”. **g** Demonstration the potential of large-scale production for films

The fabrication process of the uniform and structurally stable S-XM film framework is illustrated in Fig. 1a. Briefly, DCP was introduced into XSBR as a vulcanizing agent, followed by mixing with MXene at varying ratios and sequential blade coating. Finally, the films were hot-pressed to obtain the S-XM film. The gradient distribution of MXene endows the film framework with efficient EMI shielding, photothermal, and electrothermal capabilities. By employing the S-XM film as a universal platform for sensor integration and encapsulation, real-time monitoring of human body temperature and humidity can be achieved, thereby enabling an intelligent feedback mechanism for personalized thermal management (Fig. 1b). MXene nanosheets were synthesized by selectively etching the Al layer from the MAX (Ti_3_AlC_2_) phase (Note S1, Fig. S1) [25]. X-ray diffraction (XRD) patterns confirmed the thorough removal of Al (Fig. S2) [26]. Transmission electron microscopy (TEM) and atomic force microscopy (AFM) images (Figs. S3 and S4) further revealed the successful preparation of exfoliated MXene nanosheets with varying lateral dimensions [27]. The cross section of the S-XM film framework was characterized by scanning electron microscopy (SEM) and energy-dispersive X-ray spectroscopy (EDS), revealing a distinct layered structure with the middle layer composed of highly oriented and densely packed Ti_3_C_2_T_x_ MXene nanosheets (Figs. S5 and S6). As shown in Figs. 1c and S7, wide-angle X-ray diffraction (WAXD) analyses reveal an orientation factor (*f*) of 0.68 for the intermediate layer, exceeding that of the outer layer (0.58). Remarkably, the *f* of the assembled S-XM film reaches 0.79, indicating that the film maintains a high degree of structural alignment throughout the assembly process (Fig. 1d). The S-XM film cross section was characterized using TEM and EDS mapping. TEM images of the outer layer (Fig. S8a_1_-a_4_) and the middle layer (Fig. S8b_1_-b_4_) both show that the MXene nanosheets exhibit an oriented structure, with the interlayer spacing in the middle layer being significantly smaller than that in the outer layer. Elemental mapping of C, O, and Ti further confirms the oriented arrangement of the MXene nanosheets (Fig. S8c_1_-c_4_).

The chemical structure of the S-XM film was characterized using Fourier-transform infrared spectroscopy (FTIR) and X-ray photoelectron spectroscopy (XPS). In the FTIR spectra, the -OH stretching vibration peak of the S-XM film exhibited a red shift relative to that of the MXene film (Fig. S9) [28, 29]. Further insights were obtained from high-resolution O 1*s* XPS spectra of MXene and S-XM film surfaces (Figs. 1e and S10a), where the C–O and C=O peaks in the S-XM film were found to shift to higher binding energies. This trend was corroborated by corresponding changes in the C 1*s* spectra (Fig. S10b, c), collectively confirming the formation of hydrogen bonds between MXene and XSBR within the film framework [30]. As a result of these hydrogen bonding interactions and the rationally engineered layered architecture of the S-XM film, the material exhibits markedly improved mechanical strength while preserving excellent flexibility and foldability. Notably, as the MXene content in the outer layers increase, the tensile strength of the S-XM films rises significantly from 3.37 MPa (S-XM_0_) to 20.58 MPa (S-XM_25_), as shown in Fig. S11. In addition, the films can be folded into complex configurations, such as a “paper crane” or “spring,” without fracturing, demonstrating both macroscopic structural integrity and adaptability for wearable applications (Fig. 1f). Additionally, Fig. 1g demonstrates the large-area S-XM film fabricated using the blade coating method, which offers high scalability in layered structural design. During the coating process, it is essential to ensure uniform dispersion of the coating solution and minimize defects. By optimizing the coating parameters and selecting a polypropylene (PP) plate with good interfacial adhesion to the coating solution, the integrity of the film is maintained. The coating solution uses water-based solvents to reduce solvent costs and align with green chemistry principles, providing environmental benefits. These factors ensure the potential for stable and scalable manufacturing of this technology under basic laboratory conditions.

### Electromagnetic Interference Shielding

**Fig. 2:**
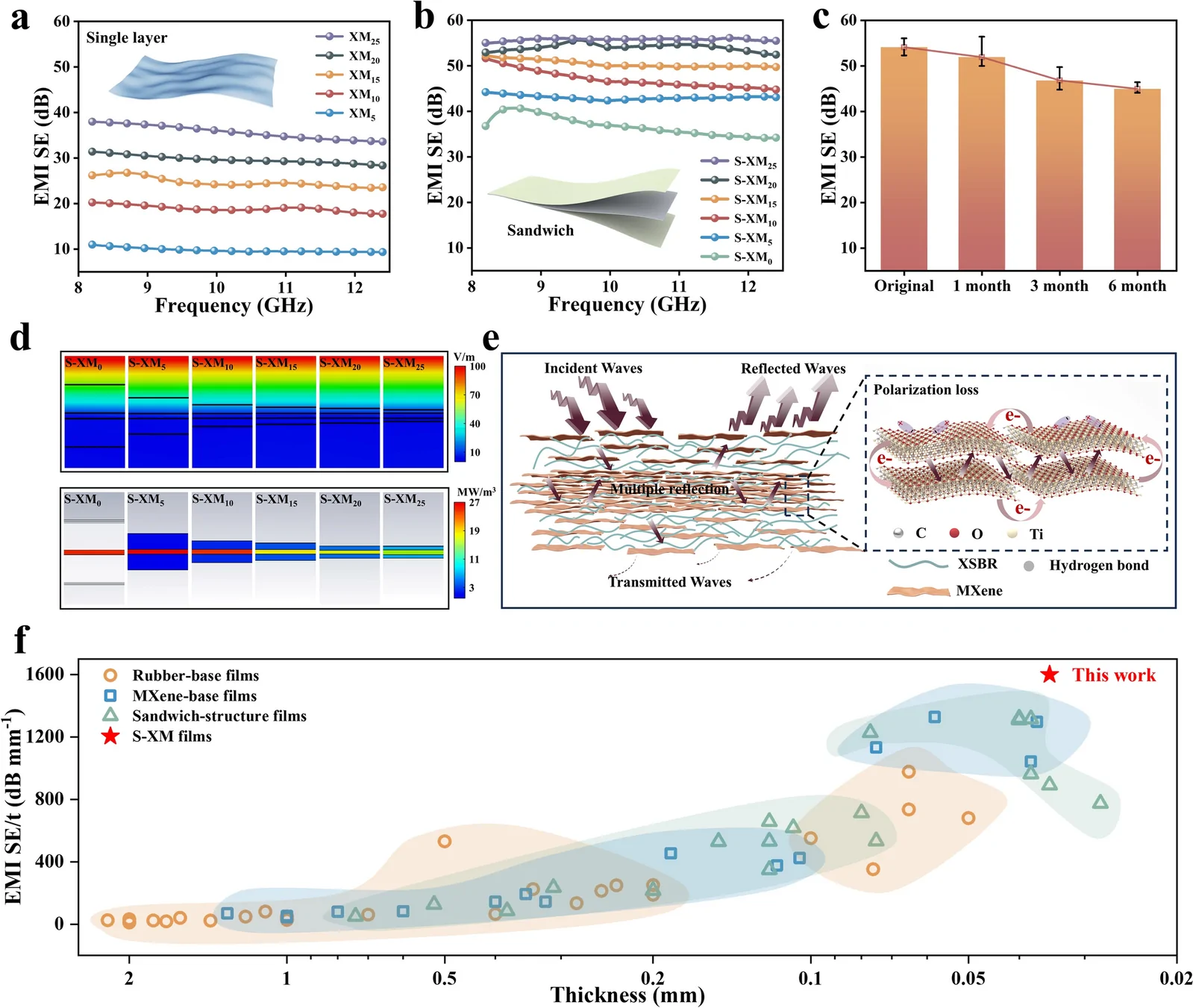
Electromagnetic interference shielding properties analysis and comparison of composite films. **a**-**b** EMI SE of single-layer XM films and S-XM films. **c** Comparison of EMI SE of S-XM Film after different exposure times without vacuum protection. **d** Electric field intensity and energy loss of S-XM films with varying outer layer MXene content. **e** EMI shielding mechanism diagram of the S-XM film framework. **f** Comparison of EMI SE/t and thickness between the S-XM film and other studies

The wearable thermal management system demonstrates exceptional EMI shielding capabilities, ensuring stable signal interaction and reliable communication. The EMI shielding performance of single-layer XM films was first evaluated within the frequency range of 8.2 to 12.4 GHz (X-band). Among them, the XM_15_ film achieved an EMI shielding effectiveness (EMI SE) of 24.7 dB, surpassing the industry standard of 20.0 dB [31]. The XM_25_ film exhibited the highest performance, with an EMI SE of 35.7 dB. Overall, the EMI SE of single-layer XM films increased progressively with higher MXene content (Figs. 2a and S12). Compared with the single-layer XM films, the S-XM film framework achieves further optimization in EMI shielding performance. Electrical conductivity is a key parameter in evaluating the electromagnetic shielding performance of materials. Conductivity measurements of the S-XM films revealed a clear upward trend with increasing outer layer MXene content. Notably, the S-XM_20_ film reaches a conductivity of 166.7 S cm^−1^ (Fig. S13). This enhancement is primarily attributed to the formation of denser and more continuous conductive networks within the film, which facilitate more efficient electron transport and improve the overall electrical conductivity. The trend of increasing conductivity is in agreement with the measured EMI SE of S-XM films in the X-band, as EMI SE increases with MXene content in the outer layer. Specifically, the EMI SE of the S-XM_0_ film (with XSBR as the outer layer) is 37 dB, while the EMI SE for the S-XM_20_ and S-XM_25_ films is 53 and 56 dB, respectively (Fig. 2b). The EMI shielding performance of the S-XM films was evaluated after storage at room temperature for different durations. The results showed that although the EMI SE exhibited a slight decline with prolonged exposure time, it remained above 45 dB (Fig. 2c). Additionally, the S-XM_20_ film maintains stable EMI shielding performance after exposure to various solvents and long-cycle bending (Figs. S14 and S15). Under these stringent tests, the S-XM film demonstrates relatively stable EMI shielding reliability. Building upon the S-XM_20_ film, a five-layer XM film (F-XM) was further fabricated using blade coating. A systematic comparison of EMI SE was conducted among the single-layer XM, S-XM_20_, and F-XM_20_ films. The F-XM_20_ film exhibited an impressive EMI SE of 70 dB at a thickness of 65 μm. This outstanding performance confirms that the EMI SE of XM films can be effectively tuned by modulating film thickness, which offers a highly adaptable solution for EMI shielding in multipurpose applications (Fig. S16).

To analyze the EMI shielding mechanism and understand the trend of EMI SE, Figs. S17 and S18 represent the average values of the reflection effectiveness (SE_R_), absorption effectiveness (SE_A_), and total shielding effectiveness (SE_T_) for the S-XM film in the X-band, along with the corresponding reflection coefficient (*R*), absorption coefficient (*A*), and transmission coefficient (*T*). Although Fig. S17 shows that SE_A_ is always higher than SE_R_, this does not imply that the EMI shielding mechanism of the S-XM_20_ film is primarily absorption-based. The reason is that SE_A_ and SE_R_ do not directly reflect the actual levels of reflection and absorption. Therefore, using the R and A to evaluate the shielding material’s performance and protective mechanism is more reasonable and intuitive [32, 33]. As shown in Fig. S18, the R of the S-XM film is much higher than the A, indicating that reflection plays a dominant role in the shielding process. The electromagnetic wave dissipation mechanisms within the S-XM film were analyzed using finite element simulations (Note S5). Figure 2d illustrates the simulated electric field intensity and energy loss on the cross section of the S-XM films with different outer layer MXene contents. As electromagnetic waves (EMWs) interact with the film surface, the color change in the electric field strength indicates that, with increasing MXene content in the outer layer, the reflection of the EMWs is enhanced. Subsequently, the electric field strength within the film gradually weakens. The transition from red to blue illustrates the dissipation of EMWs within the film, with the energy loss in the highly conductive intermediate layer being more pronounced than in the outer layer. Finite element simulations of different layered structures clearly demonstrate the stepwise dissipation of EMWs (Fig. S19). Figure 2e illustrates the EMI shielding mechanism of the S-XM film. When incident EMWs strike the surface of the film, due to significant impedance mismatch, the incident waves are primarily reflected at the interface between the S-XM film and air [34]. The EMWs that penetrate into the S-XM film are mostly dissipated through the following effects: First, internal multiple reflections and scattering occur at the heterogeneous interfaces between the MXene nanosheets and XSBR matrix, as well as between layers with differing properties. This increases the transmission path of the EMWs, enhancing their absorption [35]. Second, in the highly oriented MXene nanosheet-based conductive network, the EMWs interact with the high-density charge carriers (electrons and holes), generating a large induced current that converts electromagnetic energy into heat, resulting in ohmic losses [36, 37]. Third, local dipoles are formed between the electronegative functional groups (–F,=O, and –OH) on the MXene surface, leading to dipole polarization. The synergistic effect of the layered structure and the conductivity mismatch between MXene and XSBR further amplifies the charge storage capacity, thus enhancing interface polarization [38, 39]. Moreover, we used a reliable parameter (EMI SE/t), defined as the EMI SE divided by thickness (*t*), to evaluate the overall EMI shielding performance of S-XM films. The S-XM film achieved an EMI SE/t value as high as 1600 dB mm^−1^. Compared with previously reported rubber-based, MXene-based, and sandwich-structured EMI shielding films (Table S2), the S-XM films occupy a superior position in the upper right region of the comparison plot (Fig. 2f), highlighting their outstanding overall EMI shielding performance.

### Personal Passive Thermal Management

**Fig. 3:**
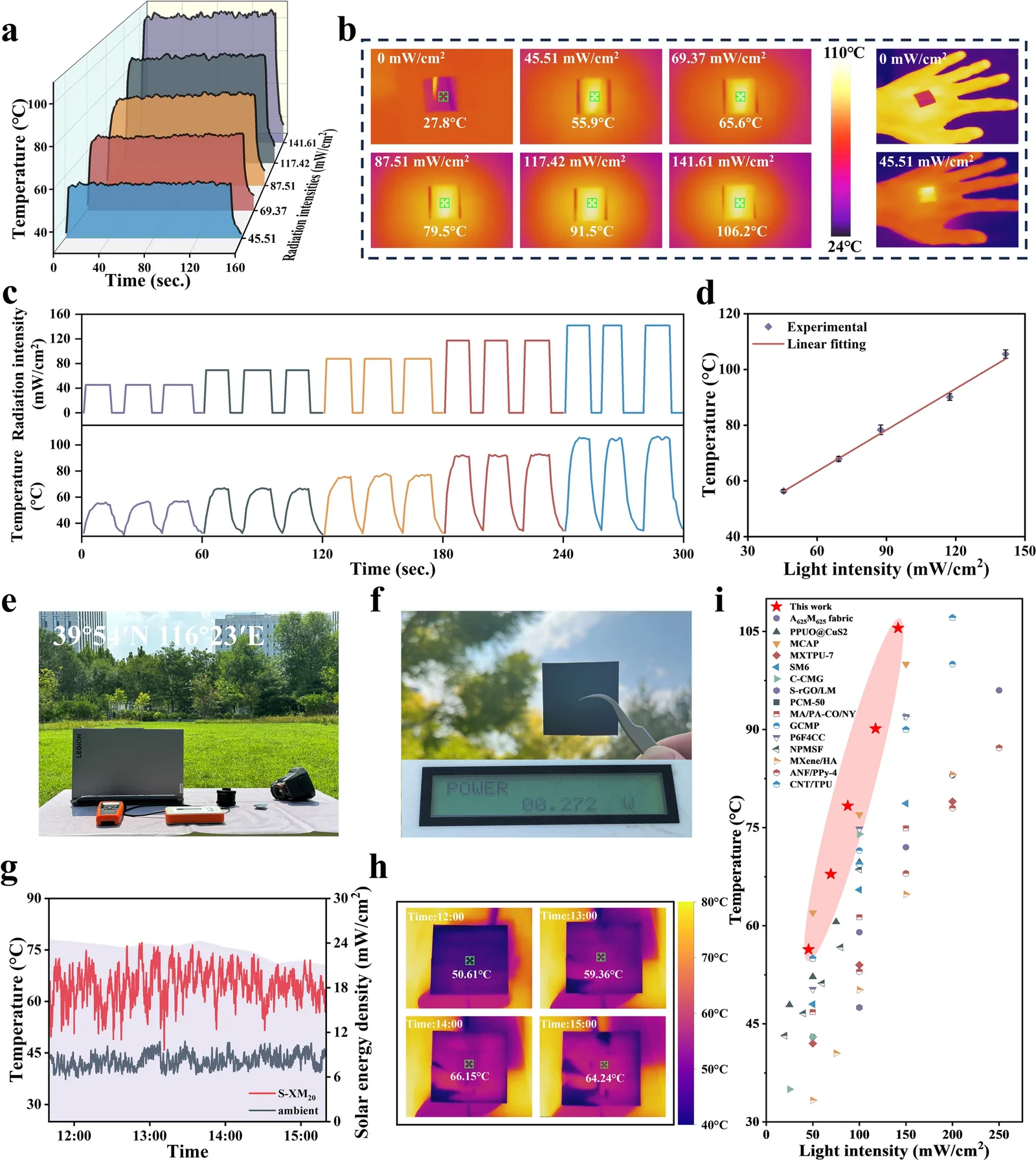
Analysis and comparison of photothermal properties of composite films. **a** Temperature profiles of S-XM_20_ film under varying radiation intensities. **b** Infrared thermal images of the S-XM film at steady state under various radiation intensities and its visualized thermal response during wearable photothermal therapy. **c** Temperature profiles of film during switching cycles with sequentially increasing radiation intensities. **d** Linear fitting for the saturation temperature versus radiation intensity. **e** Schematic diagram of outdoor photothermal test. **f** Outdoor setup for photothermal testing, showing the film and the measurement of irradiation intensity with an optical power meter. **g** Temperature variations between the S-XM_20_ film surface and the surrounding environment, as well as changes in radiation intensity, monitored during the outdoor testing. **h** Infrared thermal images of the S-XM_20_ film at different time points during outdoor photothermal performance testing. **i** Comparison of the photothermal performance of the S-XM_20_ film with results reported in previous studies

Under complex environmental conditions, the flexible wearable thermal management system with multi-module collaborative interaction ensures reliable personal thermal regulation, effectively preventing the risk of hypothermia. The S-XM_20_ film, incorporating MXene, exhibits outstanding photothermal conversion capabilities [40]. As shown by the solar absorption spectra of XSBR and S-XM_20_ films in the ultraviolet–visible–near-infrared (UV–vis–NIR) range (0.2 ~ 2.5 μm) (Fig. S20), the S-XM_20_ film exhibits an average absorbance of 92.09%, whereas the XSBR film shows significantly lower overall absorption. When exposed to sunlight, the free electrons on the surface of MXene undergo collective oscillation, enhancing the local electric field and significantly boosting light absorption, which is subsequently converted into thermal energy [41]. In addition, the layered structure of the S-XM_20_ film facilitates multiple reflection and scattering of incident light, further improving its light-harvesting capability [42]. To systematically evaluate the photothermal performance of the S-XM_20_ film, a xenon lamp was employed to simulate sunlight. The S-XM_20_ film rapidly (< 5 s) reaches the corresponding steady-state temperature under varying radiation intensities (45.51 ~ 141.61 mW cm^−2^). Notably, even at a low irradiation intensity of 45.51 mW cm^−2^, the surface temperature of the film reached as high as 56.38 °C (Fig. 3a). Moreover, the background plate has a negligible effect on the photothermal performance of the film (Fig. S21). Infrared thermal images further demonstrate a uniform temperature distribution across the entire surface of the film, indicating its potential for stable heat supply when applied to human skin (Fig. 3b). The high sensitivity and stability of the photothermal performance enable thermal management to be more advantageous. The dynamic photothermal response of the S-XM_20_ film was evaluated (Fig. 3c), which demonstrated rapid response times during both heating and cooling processes, as well as excellent stability during cyclic operations. Furthermore, the S-XM_20_ film maintained stable performance after 150 heating–cooling cycles (with radiation intensity ranging from 0 to 45.51 mW cm^−2^), with no significant degradation in its photothermal properties (Fig. S22). After treatment at 60% RH for 24 h (simulating human skin humidity) and multiple bending cycles, the film continued to achieve rapid temperature response under a radiation intensity of 45.51 mW cm^−2^. The steady-state temperature did not show significant decline when compared to the untreated sample (Fig. S23). By performing linear fitting on the steady-state temperatures achieved under different radiation intensities, the linear regression coefficient (*R*^*2*^) was found to be as high as 0.99. This indicates that the S-XM_20_ film exhibits tunable and predictable photothermal performance capabilities (Fig. 3d).

The photothermal performance of the S-XM_20_ films in real outdoor environments requires further evaluation. We conducted a test on July 17, 2024, in an outdoor open environment (Fig. 3e), using a power meter to monitor the solar power density (Figs. 3f and S24). Between 11:30 and 15:30, we used a contact thermocouple to record the surface temperature of the S-XM_20_ film as well as the ambient temperature. The S-XM_20_ film exhibited a significant temperature increase when irradiated and was able to maintain stable heating over an extended period, although slight temperature fluctuations were observed due to wind interference in the outdoor test environment (Fig. 3g). Infrared thermal images were taken at 12:00, 13:00, 14:00, and 15:00 (Fig. 3h), and the temperature readings were consistent with those measured by the thermocouple. The S-XM_20_ film exhibits a high response speed, low energy consumption, and high light energy absorption efficiency, surpassing the performance of some photothermal film materials previously reported in literature (Fig. 3i and Table S3).

### Personal Active Thermal Management

**Fig. 4:**
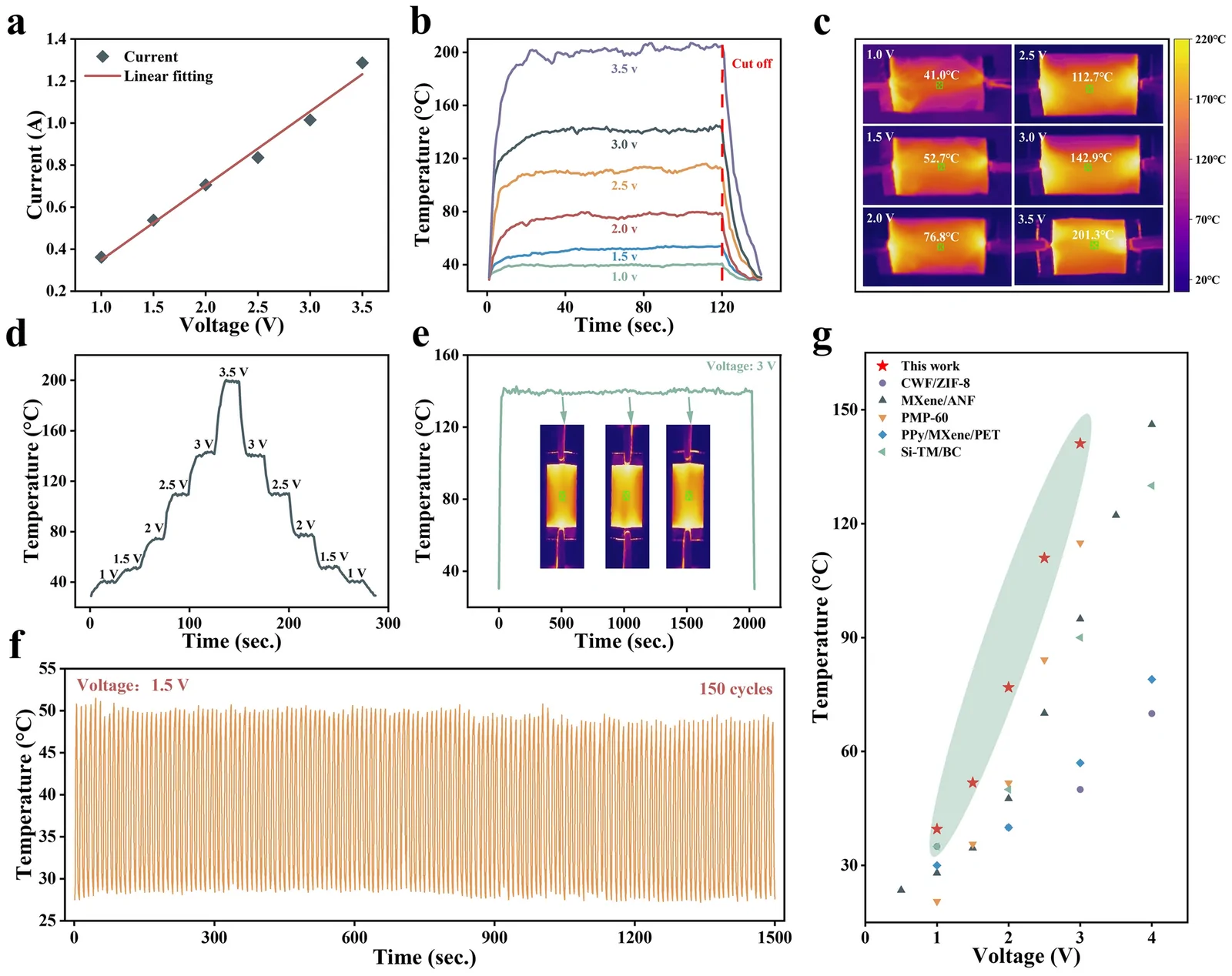
Analysis and comparison of Joule heating of composite films. **a** V-I linear curve of S-XM_20_ film. **b** Surface temperature profiles of the S-XM_20_ film under different applied DC voltages. **c** Infrared thermal images of the S-XM_20_ film under different applied DC voltages. **d** Temperature variation curve on the surface of the S-XM_20_ film during dynamic voltage adjustment. **e** Stability test of prolonged Joule heating (3 V for 2000 s). **f** Temperature curve of the S-XM_20_ film over 150 switching cycles (with the driving voltage varying from 0 to 1.5 V). **g** Comparison of the Joule heating performance of the S-XM_20_ film with results reported in previous studies

Although the S-XM_20_ film exhibits excellent photothermal properties, enabling passive thermal management under ample sunlight, the flexible wearable thermal management system must possess active temperature regulation capabilities in conditions of low solar irradiance or during nighttime. Experimental results show reveal the corresponding current values, within the 1.0 – 3.5 V direct current (DC) voltage range (Fig. 4a). The fitted V-I linear curve demonstrates a high degree of correlation (0.9846), indicating that the Joule heating of this composite film largely obeys Ohm’s law, making it a reliable electric heater [3]. Notably, the S-XM_20_ film generates a current of 0.538 A under a 1.5-V drive, with its surface temperature reaching a steady-state value of 51.79 °C at this voltage (Fig. 4b). Infrared thermal imaging further confirms that, at each applied voltage, the temperature distribution across the entire film is uniform, with no macroscopic structural damage observed (Fig. 4c). The film’s temperature shows a linear relationship with the square of the applied voltage, in accordance with Joule’s law ($Q = U^{2} R^{ - 1} t$) [43], indicating that the S-XM_20_ film exhibits excellent controllability and adjustability for active thermal management applications (Fig. S25).

In practical thermal management applications, the reliability testing of the flexible wearable thermal management system is crucial. The S-XM_20_ film demonstrates the ability to rapidly reach a steady-state temperature under dynamic voltage switching conditions, highlighting its feasibility in handling rapid temperature fluctuations typical of real-world environments (Fig. 4d). During 150 low-voltage switching cycles (voltage range: 0 – 1.5 V), the S-XM_20_ film exhibited excellent cycling stability, with rapid temperature response during both heating and cooling, while maintaining a consistent steady-state temperature throughout the extended cycles (Fig. 4f). Additionally, under higher voltage switching cycles (voltage range: 0 – 3 V), the film also maintained stable heating and cooling behavior (Fig. S26). Long-term stability is a critical parameter for evaluating the reliability of the S-XM_20_ film in thermal management applications. Under a constant voltage of 3 V, the surface temperature of the film remained stable for 2000 s. Infrared thermal images captured at various time intervals further verified the film’s capability to deliver stable and continuous heat output (Fig. 4e). Furthermore, we evaluated the Joule heating performance of the material after treatment simulating the humidity conditions on human skin and repeated mechanical bending. The results indicate that, although the steady-state temperature slightly decreased after treatment, the Joule heating response remained rapid, and stable heating was still achieved (Fig. S27). We summarized and compared the Joule heating performance reported in literature, revealing that the S-XM_20_ film achieves higher temperatures under the same applied voltage. This demonstrates its superior active thermal management capability and lower energy consumption (Fig. 4g and Table S4). The excellent synergy between active and passive thermal management enables flexible adaptation to various complex environments. By applying a 1.5-V voltage to the S-XM_20_ film and simulating sunlight using a xenon lamp (radiation intensity: 45.51 mW cm^−2^), a synergistic heating mode combining Joule heating and photothermal effects was established. At 1.5 V, the steady-state surface temperature of the film reached 48.3 °C. Upon exposure to simulated sunlight, the surface temperature of the film rapidly increased and stabilized at 88.6 °C (Fig. S28). This result demonstrates the feasibility of the dual-mode synergistic heating system, significantly enhancing the versatility of its thermal management functionality.

### Bimodal Temperature–Humidity Sensing

**Fig. 5:**
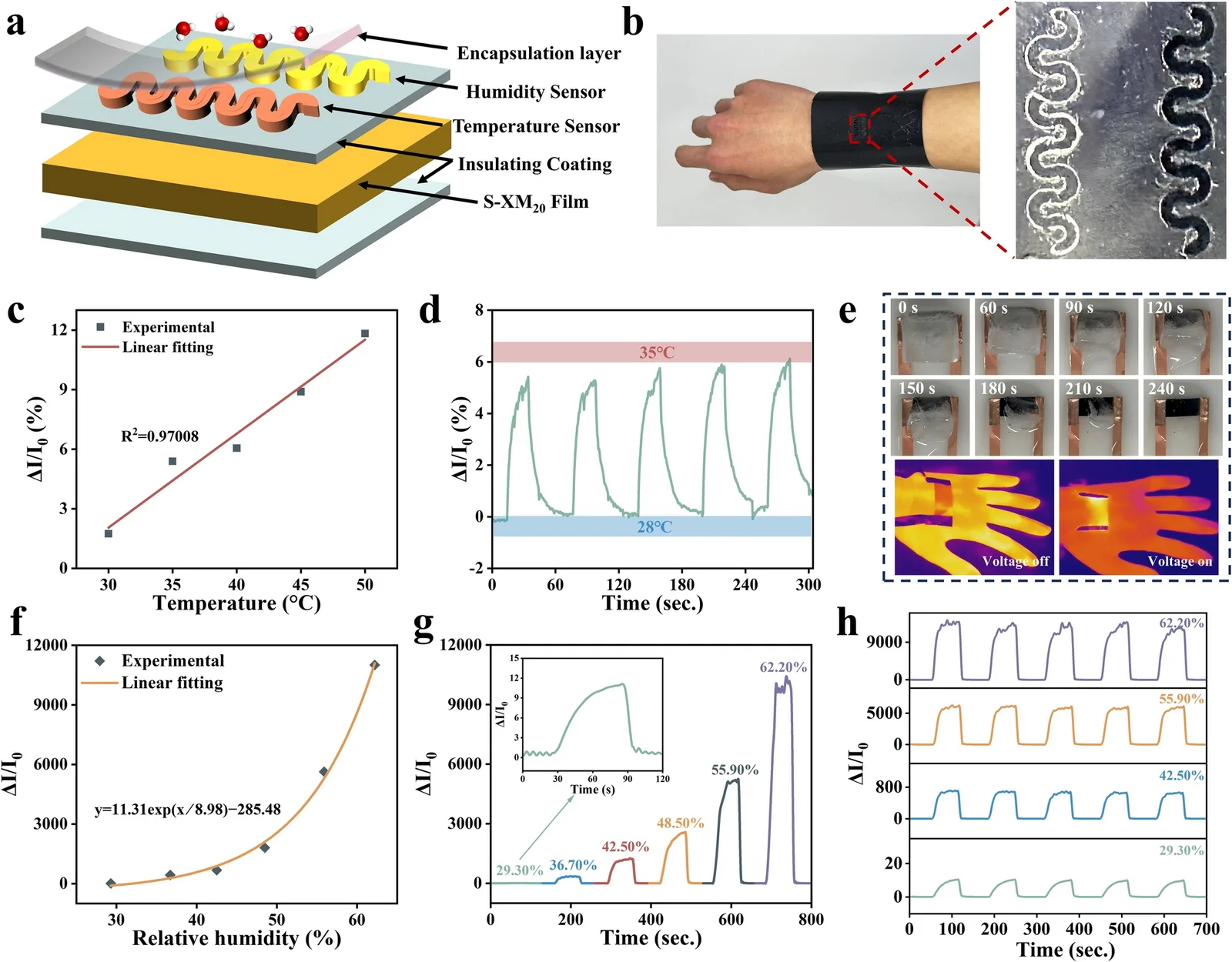
Analysis and comparison of dual-mode temperature–humidity sensing properties of composite films. **a** 3D schematic of the flexible wearable sensor design, showing the encapsulation layer, serpentine patterned temperature–humidity sensor, insulating layer, and S-XM_20_ film. **b** Schematic of a flexible wearable XM film integrated with a temperature–humidity sensor. **c** Relationship between the normalized relative current change of the temperature sensor and temperature. **d** Electrical signal response of the sensor when a low-temperature signal is detected, followed by heating the film to 35 °C. **e** Functional synergy of Joule heating and temperature sensing technologies for combined de-icing and heating processes. **f** Dynamic response curve of the humidity sensor at varying RH levels. **g** Relationship between the normalized relative current change of the humidity sensor and relative humidity, ranging from 29.30% to 62.20%. **h** Stability test of the humidity sensor at RH values of 29.30%, 42.50%, 55.90%, and 62.20%

With the advancement of flexible electronics and artificial intelligence (AI) algorithms, human–machine interaction (HMI) has undergone a paradigm shift [44, 45]. Integrating temperature and humidity sensing capabilities into wearable devices is crucial for intelligent thermoregulation, which benefits user comfort and safety [46]. The fabrication process of the bimodal temperature–humidity sensor fully utilizes the advantages of a layer-by-layer design. Temperature–humidity sensors were integrated onto S-XM_20_ films framework pre-coated with an insulating layer via a masking technique, and subsequently encapsulated. The complete structural configuration of the sensor is illustrated in Fig. 5a. Careful modeling and design of sensing materials are critical in ensuring compatibility between sensors and the human body [47]. To enhance comfort, a serpentine structure was employed in the sensor design, characterized by relatively low bending stiffness, which contributes to improved mechanical stability. This structural configuration effectively mitigates strain-induced degradation, promotes better adhesion to the skin, and enhances the reliability of health monitoring [48, 49]. Laser micro-manufacturing technology enables high-precision processing of various materials and high-throughput production of complex structures, greatly facilitating the temperature–humidity sensor structure design [50]. Moreover, the temperature and humidity sensors occupy a minimal area in the S-XM_20_ film (Fig. 5b), making this design a promising solution for the development of distributed multi-sensor systems. The encapsulation has a negligible effect on the conductivity and electromagnetic shielding performance of the S-XM_20_ layer. However, due to the difference in elastic moduli between the XSBR layer and the conductive S-XM_20_ layer, stress-induced strain distribution becomes uneven, leading to stress concentration and a slight decrease in the mechanical performance of the film (Fig. S29).

The S-XM_20_ film, integrated with temperature and humidity sensors, demonstrates the potential of an intelligent monitoring and feedback-driven thermal management system. When external environmental changes trigger a hypothermia risk, the temperature and humidity sensor within the film detects the variation, converts it into an electrical signal, and transmits it to the microcontroller unit (MCU). After signal processing, the MCU evaluates the hypothermia risk and activates the heating function. Once the temperature reaches the set value, the MCU cuts off the voltage supply, halting the heating process and ensuring that the temperature remains stable within the target range. This mechanism enables automatic adjustment and temperature regulation through closed-loop control, ensuring stable system operation (Fig. S30). However, we recognize that current research is primarily focused on the material level. Achieving system-level wearable thermal management applications still faces a multitude of challenges, particularly in back-end circuit control, sensor signal processing, precise feedback mechanism regulation, and heating system efficiency optimization. Although experimental results validate the basic activation mechanisms of the sensors and heaters, further improvements in system design and optimization are needed to realize a fully integrated, real-time responsive intelligent system.

For the temperature sensing material, poly(3,4-ethylene dioxythiophene): poly(styrenesulfonate) (PEDOT:PSS) was selected due to its high conductivity, mechanical flexibility, ease of processing, and excellent compatibility with a wide range of substrates [51, 52]. Within the comfortable human body temperature range (30 ~ 50 °C), the sensor exhibits outstanding linearity in its temperature response, with *R*^*2*^ of 0.97 (Fig. 5c). Figure 5d demonstrates that when the sensor detects a body temperature below the normal range, active thermal management is initiated to heat the S-XM_20_ film, thereby raising the temperature to a comfortable level. The sensor also demonstrates excellent stability during heating and cooling cycles between 28 and 35 °C. Its response at various temperatures shows both stability and high reproducibility in *ΔI/I₀* over five cycles (Fig. S31). The integration of temperature sensors with the thermal management system endows the S-XM_20_ film with exceptional practical application potential. As shown in Figs. 5e and S32, an ice block was placed on the surface of the S-XM_20_ film to simulate icing under extreme environmental conditions. Upon detecting a sudden drop in temperature, the temperature sensor quickly generates an electrical signal and transmits it to the film thermal management system. The system then activates the Joule heating mechanism, raising the film temperature to rapidly remove the ice, while simultaneously providing effective thermal protection for the human body. Furthermore, infrared thermal images were used to visually demonstrate the S-XM_20_ film ability to continuously and stably supply heat when applied to the surface of human skin, further confirming its potential for use in wearable devices (Fig. S33).

Highly sensitive, efficient, and stable humidity sensors provide enhanced support for regulating human thermal comfort. To achieve humidity detection in wearable devices, we developed a humidity sensor by incorporating KOH into a polyvinyl alcohol (PVA) matrix. KOH exhibits significantly higher solubility in water compared to PVA. During the moisture adsorption process, K^+^ and OH^−^ migrate from the PVA matrix into the adsorbed water, forming a liquid electrolyte [53]. The directional migration of these ions under an electric field substantially increases the number of charge carriers in the water layer, thereby altering the electrical signal output [54, 55]. As illustrated in Fig. 5f, the sensor response (expressed as △*I/I*_0_) shows an exponential dependence on relative humidity (RH), which can be fitted using the following equation: $y = 11.31e^{x/8.98} - 282.48$, with *R*^*2*^ of 0.99. Furthermore, the sensor demonstrates a broad detection range (20% ~ 70%). Figure 5g illustrates the dynamic response curve of the PVA/KOH humidity sensor. The sensor exhibits a sensitive response during the dynamic increase in humidity, significantly demonstrating its stability and sensitivity. As shown in Fig. 5h, the sensor exhibits high reproducibility across five cycles at various RH levels (29.30%, 42.50%, 55.90%, and 62.20%).

## Conclusions

In this work, we developed a hierarchical modular design strategy to construct a multi-module synergistic flexible wearable thermal management system, realizing a “monitoring-feedback-protection” closed-loop mechanism. The wearable thermal management system features highly stable, sensitive, and low-power electrothermal (51.79 °C at 1.5 V) and photothermal (56.38 °C at 45.51 mW cm^−2^) properties, enabling reliable back-end collaborative heating. The integrated biomimetic serpentine dual-mode sensor offers a wide detection range, further enhancing the system’s front-end temperature and humidity monitoring capabilities. When coupled with the electro/photothermal conversion module, it enables intelligent thermal regulation, significantly improving adaptability to complex environments, including efficient de-icing under extreme cold conditions. The system also demonstrates excellent EMI shielding performance, achieving an EMI SE/t value of 1600 dB mm^−1^ at a thickness of just 35 μm, while ensuring stable signal interaction. Overall, this strategy provides a practical and scalable pathway for the multifunctional integration and performance optimization of next-generation flexible wearable electronics.

## Supplementary Information

Below is the link to the electronic supplementary material.

Supplementary file1 (DOCX 15558 kb)
