# Supplementary material for: Hierarchical Modular Architecture Enabling Intelligent Dynamic Thermal Management and Superior Electromagnetic Interference Shielding
Source: Nanomicro Lett. 2026 Mar 18;18:290. doi: 10.1007/s40820-026-02140-9 (PMC12996553; doi:10.1007/s40820-026-02140-9)
Supplement: Supplementary file 1 — Supplementary file1 (DOCX 15558 kb) [file 40820_2026_2140_MOESM1_ESM.docx]

Supporting Information for

**Hierarchical Modular Architecture Enabling Intelligent Dynamic Thermal Management and Superior Electromagnetic Interference Shielding**

Qi-Fan Xuan^1,2^, Pei-Yan Zhao^1^, Hualong Peng^1,2^, Shan Zhang^1,2^, Bo Cai^1,2^, Fang-Yu Niu^2^, Martin C. Koo^1,2^, Xiao-Bo Sun^2^, Xiangyu Jiang^2^* and Guang-Sheng Wang^1,2^*

^1^State Key Laboratory of Bioinspired Interfacial Materials Science, Bioinspired Science Innovation Center, Hangzhou International Innovation Institute, Beihang University, Hangzhou 311115, P. R. China

^2^School of Chemistry, Beihang University, Beijing 100191, P. R. China

*Corresponding authors. E-mail: [jiangxy@buaa.edu.cn](mailto:jiangxy@buaa.edu.cn) (Xiangyu Jiang); [wanggsh@buaa.edu.cn](mailto:wanggsh@buaa.edu.cn) (Guang-Sheng Wang)

**Note S1 Preparation of (Ti_3_C_2_T_x_) MXene nanosheets**

Titanium Carbide (Ti_3_C_2_T_x_) MXene nanosheets were obtained by selective etching of Al element from MAX (Ti_3_AlC_2_) powder with HCl and LiF. Firstly, the LiF (2.0 g) powder and HCl (40 mL 9M) were added to a Teflon vessel and stirred for 10 min at room temperature. Subsequently, 2.0 g of Ti_3_AlC_2_ powder was slowly added to the etching solution over a period of more than 3 min. The reaction was carried out at 650 r/min in a water bath at 35 °C for 24 h. After the reaction, the mixture was centrifuged at 3,500 rpm for 10 min, and the precipitate was washed with deionized water until the pH~6. The resulting suspension was sonicated for 30 min in an ice-water bath under an argon atmosphere. Finally, the treated solution was centrifuged again at 3,500 rpm for 3 min to obtain a homogeneous Ti_3_C_2_T_x_ suspension. To achieve a concentrated Ti_3_C_2_T_x_ suspension, the solution was centrifuged at 10,000 rpm for 40 min. The supernatant was discarded, and a suitable amount of water was added to obtain a concentrated Ti_3_C_2_T_x_ suspension with a final concentration of 30 mg/mL.

**Note S2 Preparation of temperature and humidity sensing materials**

The temperature sensor employs commercial PEDOT:PSS as its active material, which is homogenized with an adjustable mixer immediately before use. For the humidity sensor, a PVA/KOH hydrogel serves as the sensing layer. Specifically, 1 g of polyvinyl alcohol (PVA) was dispersed in 23.5 g of de-ionized water (4 wt % PVA) and allowed to swell for 5 min at room temperature. The mixture was then stirred at 85 °C and 500 rpm on a heated magnetic stirrer for 2 h, with the beaker covered in cling film to minimize evaporation. Separately, 3 g of KOH was dissolved in 5 mL of de-ionized water and cooled to room temperature. Once fully dissolved, 0.833 mL of the KOH solution (providing 2 wt % KOH relative to the PVA solution) was added dropwise to the PVA solution. The reaction was maintained at 85 °C and 500 rpm for an additional 30 min, after which the mixture was cooled to room temperature and stored for subsequent use.

**Note S3 Characterizations**

X-ray diffraction (XRD) curves were acquired using an X-ray diffractometer (XRD-6000, Shimadzu, Japan) with a scan range from 5° to 60° and a scan rate of 5° min^-1^. The morphologies and nanostructures were examined by field-emission scanning electron microscopy (SEM, JSM-7500F) and transmission electron microscopy (TEM, JEM-2100F). The surface morphology and structure of the material were explored using atomic force microscopy (AFM, Bruker Dimension ICON, Germany). The surface chemistry of MXene and the XSBR/MXene (XM) film was characterized using X-ray photoelectron spectroscopy (XPS, Thermo Fisher Scientific ESCALAB Xi, USA). The chemical characteristics of the material were analyzed using Fourier transform infrared spectroscopy (FTIR, Bruker VERTEX 70v, Germany) in the wavenumber range of 4000 to 600 cm^-1^.

The mechanical properties which involved fracture strength and tensile elongation were tested by a universal mechanical testing machine (AGS-X 1KN, Japan), the load-displacement and stress-strain data are transformed as follows:

$$\begin{aligned} \sigma\left( MPa \right)=\frac{F\left( N \right)}{d\left( mm \right)\times t\left( mm \right)} (S1) \end{aligned}$$

$$\begin{aligned} \varepsilon=\frac{x\left( mm \right)}{l\left( mm \right)}\times100\% (S2) \end{aligned}$$

Where *σ* denotes stress and *ε* denotes strain, *d*, *t* and *l* represent the width, thickness and the length of the sample, respectively.

The electrical conductivity and square resistance of the sample surface were measured using a four-point probe resistivity measurement system (KDB-1, China). The electrical conductivity of the composite film was then calculated as follows:

$$\begin{aligned} \sigma\left( S/cm \right)=\frac{1}{R\left( \Omega\right)\times t\left( cm \right)} (S3) \end{aligned}$$

Where 𝜎 denotes the electrical conductivity and *t* denotes the thickness of the sample.

Wide-angle X-ray scattering (WAXS) tests were conducted on a Xenocs Xeuss SAXS/WAXS system using an incident Cu-Kα X-ray beam parallel to the film plane. The distance between sample and detector is 30.00 cm. The samples for WAXS tests were 2.0 mm wide, 15 mm long strips. The scattering patterns were recorded by a PILATUS 300k detector. The alignment degree of MXene nanosheets was quantified by using the Herman’s orientation factor (*f*), which is defined as follows:

$$\begin{aligned} f=\frac{1}{2}\left( 3\left\langle\cos^{2} \emptyset\right\rangle-1 \right) (S4) \end{aligned}$$

where 〈*cos^2^ ϕ*〉 is the average value of the square of the cosine of the azimuthal angle for the (002) peak of single-layer XM and S-XM films, which is calculated as follows:

$$\begin{aligned} \left\langle\cos^{2} \emptyset\right\rangle=\frac{\int_{0}^{\pi/2} I\left( \emptyset\right)\cos^{2} \emptyset\sin\emptyset d\emptyset}{\int_{0}^{\pi/2} I\left( \emptyset\right)\sin\emptyset d\emptyset} (S5) \end{aligned}$$

where *I(ϕ)* is the intensity at an azimuthal angle of *(ϕ)*.

The UV-Vis-NIR absorption spectra of XSBR and S-XM_20_ films were measured using a UV-Vis spectrometer (Hitachi UH4150, Japan) in integrating sphere mode. This setup was employed to determine the films diffuse reflectance (*R%*) and transmittance (*T%*) across the wavelength range of 200~2500 nm, with absorptance (*A%*) calculated as follows:

$$\begin{aligned} A\%=1-R\%-T\% (S6) \end{aligned}$$

**Note S4 EMI shielding performance testing**

The electromagnetic interference (EMI) shielding performance in the frequency range of 8.2-12.4 GHz (X-band) was evaluated on a vector network analyzer (Agilent PAN-N5244A), and the sample size was 22.5×10.1 mm^2^. Two scattering parameters of electromagnetic radiation (S_11_ and S_21_) were measured by waveguide method, and the power coefficient and the EMI shielding effectiveness (EMI SE) of the XM films are calculated according to the following equations:

$$\begin{aligned} R=\left| S_{11} \right|^{2}=\left| S_{22} \right|^{2}, T=\left| S_{12} \right|^{2}=\left| S_{21} \right|^{2} (S7) \end{aligned}$$

$$\begin{aligned} A=1-R-T (S8) \end{aligned}$$

$$\begin{aligned} {SE}_{R}=-10\log\left( 1-R \right) (S9) \end{aligned}$$

$$\begin{aligned} {SE}_{A}=-10\log\left( \frac{T}{1-R} \right) (S10) \end{aligned}$$

$$\begin{aligned} {SE}_{T}=-10\log T={SE}_{R}+{SE}_{A}+{SE}_{M} (S11) \end{aligned}$$

where *R*, *A*, and *T* are the coefficients of reflectivity, absorptivity, and transmission, respectively, and *SE_T_*, *SE_R_*, *SE_A_*, and *SE_M_* are the total shielding effectiveness, microwave reflection, microwave absorption and multiple reflection, respectively. *SE_M_* can usually be ignored when *SE_T_* > 15 dB.

Furthermore, the EMI SE divided by the thickness (EMI SE/t) of can be calculated using the following equation:

$$\begin{aligned} EMI SE/t=\frac{{SE}_{T}}{t} (S12) \end{aligned}$$

Here, *t* represents the thickness of the films.

**Note S5 CST simulation of XM EMI shielding film**

The electromagnetic simulations were conducted using CST Studio Suite 2014 software. By importing the material model and integrating the conductivity parameters (specific conductivity values are provided in Table S1), the electric field distribution and energy loss distribution of the S-XM film were simulated. To optimize computational efficiency, periodic boundary conditions were applied to a single unit cell. The configuration of the electromagnetic source included periodic boundary conditions parallel to the material surface, as well as open boundary conditions perpendicular to the material surface. Mesh convergence was successfully achieved through the use of adaptive mesh refinement with tetrahedral mesh elements.

**Table S1** CST simulation parameters

|  | ***d*_top_ (mm)** | *𝛔***_top_ (S/m)** | ***d*****_middle_ (mm)** | *𝛔***_middle_ (S/m)** | ***d*_bottom_ (mm)** | *𝛔***_bottom_ (S/m)** |
| --- | --- | --- | --- | --- | --- | --- |
| **S-XM_0_** | 0.064 | 0.000 | 0.01 | 14285.714 | 0.064 | 0.000 |
| **S-XM_5_** | 0.035 | 54.745 | 0.01 | 14285.714 | 0.035 | 54.745 |
| **S-XM_10_** | 0.019 | 620.655 | 0.01 | 14285.714 | 0.019 | 620.655 |
| **S-XM_15_** | 0.014 | 1601.537 | 0.01 | 14285.714 | 0.014 | 1601.537 |
| **S-XM_20_** | 0.011 | 2336.995 | 0.01 | 14285.714 | 0.011 | 2336.995 |
| **S-XM_25_** | 0.008 | 4166.667 | 0.01 | 14285.714 | 0.008 | 4166.667 |

**Note S6 Photothermal and Joule heating performance testing**

To study the photothermal properties, the film was placed in a solar simulator (CEL-PUV300-T8, China) while the radiation intensity was monitored using an optical power meter (PL-MW2000, China). An infrared thermal camera (HIKMICRO HM-TP73-15SVF/W/4G, China) provided real-time temperature monitoring and infrared thermal imaging. For outdoor photothermal effect measurements, a dual-channel K-type thermocouple (YET-620L, China) was employed to record the temperature of the film at 1 s intervals.

The Joule heating performance of the film was assessed using a direct current (DC) power supply (MS-305DS, China) at specified voltages, while the surface temperature changes were observed and documented using an infrared thermal camera.

**Note S7 Temperature and humidity sensor performance testing**

To investigate the performance of the film temperature sensors, the films were placed in a high- and low-temperature vacuum probe stage (CGO-4, Cindbest). Probes were connected to the two ends of the serpentine sensors, and voltages were applied to the films using a DC power supply, bringing the films to various temperatures. Simultaneously, a semiconductor characterization system (4200-SCS, KEITHLEY) was employed to monitor changes in the current signal of the temperature sensors as the temperature increased. The sensitivity and current temperature coefficient of the temperature sensors were then calculated using the following equations:

$$\begin{aligned} \frac{\Delta I}{I_{0}}=\frac{\left( I-I_{0} \right)}{I_{0}} (S13) \end{aligned}$$

The humidity sensor test involved placing the film in a high- and low-temperature vacuum probe stage and connecting the probe to both ends of the serpentine sensor. The humidity conditions were regulated using a liquid dynamic dilution gas dispenser (H310V, Tianjin Huayi Science and Technology Co., Ltd.). The current signals of the humidity sensor under varying humidity levels were then detected using a semiconductor characterization system.

**Supplementary Figures**


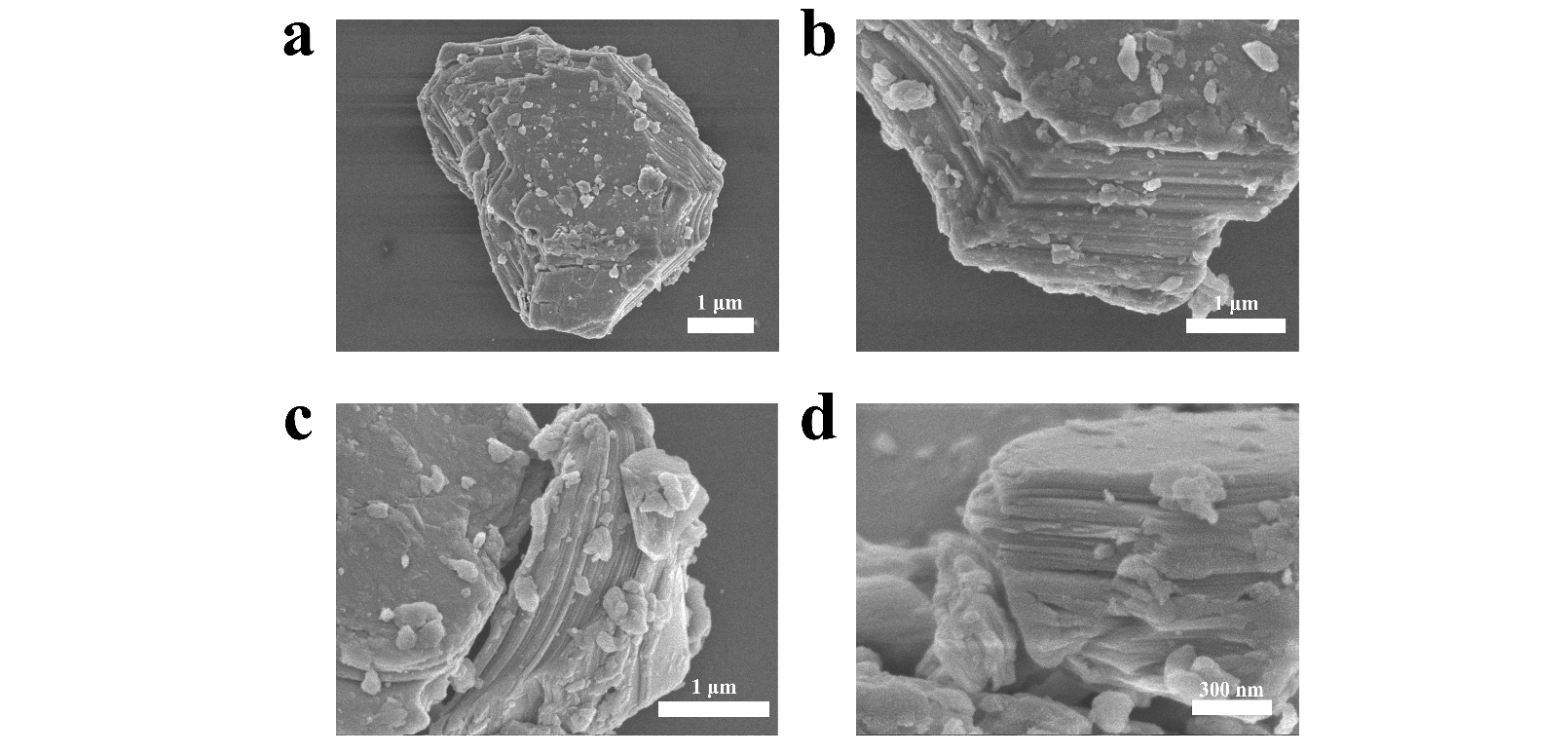


**Fig. S1** SEM images of MAX (Ti_3_AlC_2_) powder

Figure S1 illustrates the MAX (Ti_3_AlC_2_) power displays a multilayered lamellar stacking structure.


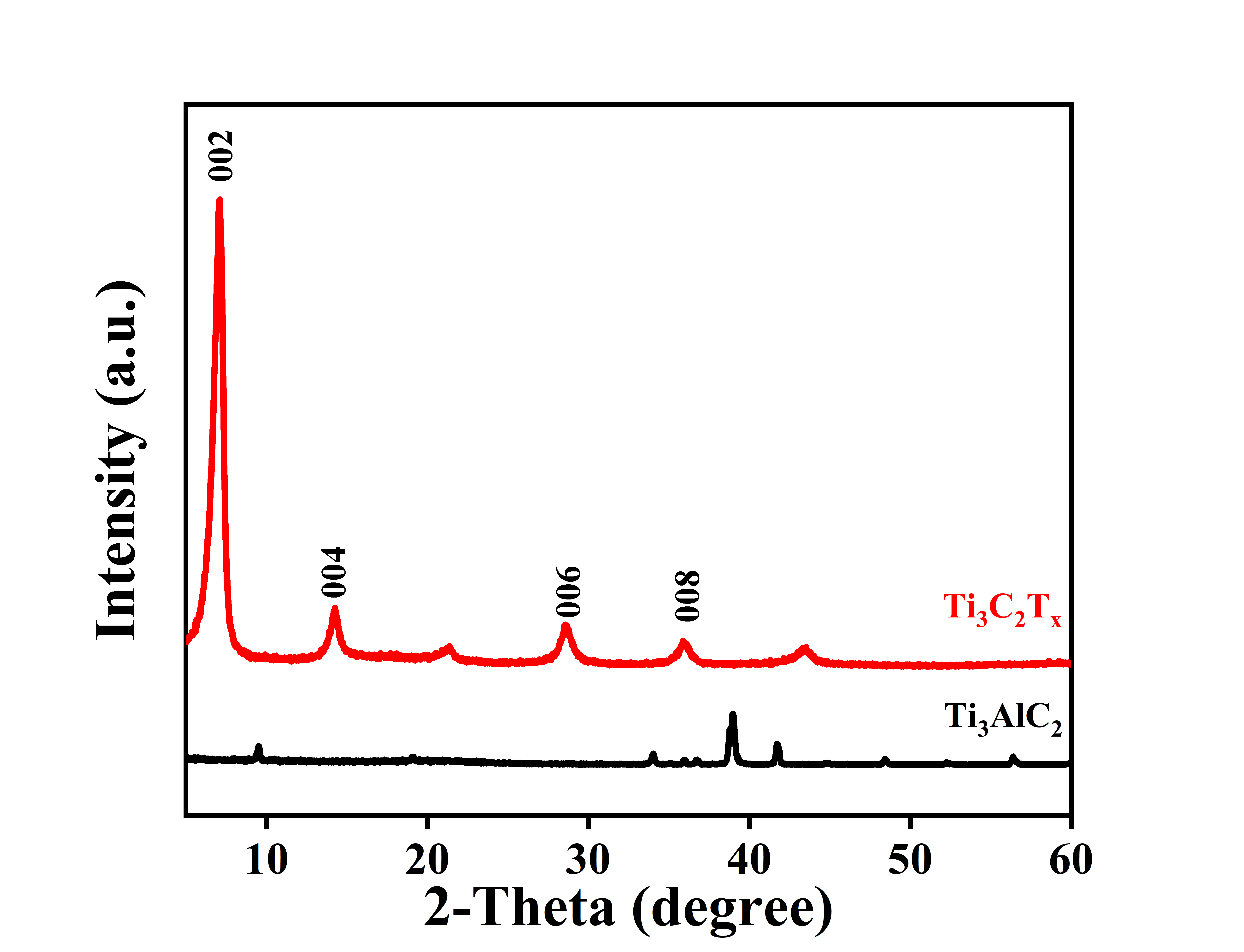


**Fig. S2** XRD spectrum of MXene (Ti_3_C_2_T_x_) and MAX (Ti_3_AlC_2_)

After etching the MAX phase with HF, the main Ti peak (002) in the XRD curve of Ti_3_C_2_T_x_ shifted from 9.52° to 7.12°, suggesting an increase in the layer spacing of the nanosheets due to etching and stripping. Additionally, characteristic peaks at 14.26°, 28.58°, and 35.88° corresponded to the (004), (006), and (008) crystal planes of MXene, respectively. The disappearance of the characteristic peak of Al at 39° indicated successful removal of the Al layer and synthesis of Ti_3_C_2_T_x_ MXene nanosheets.


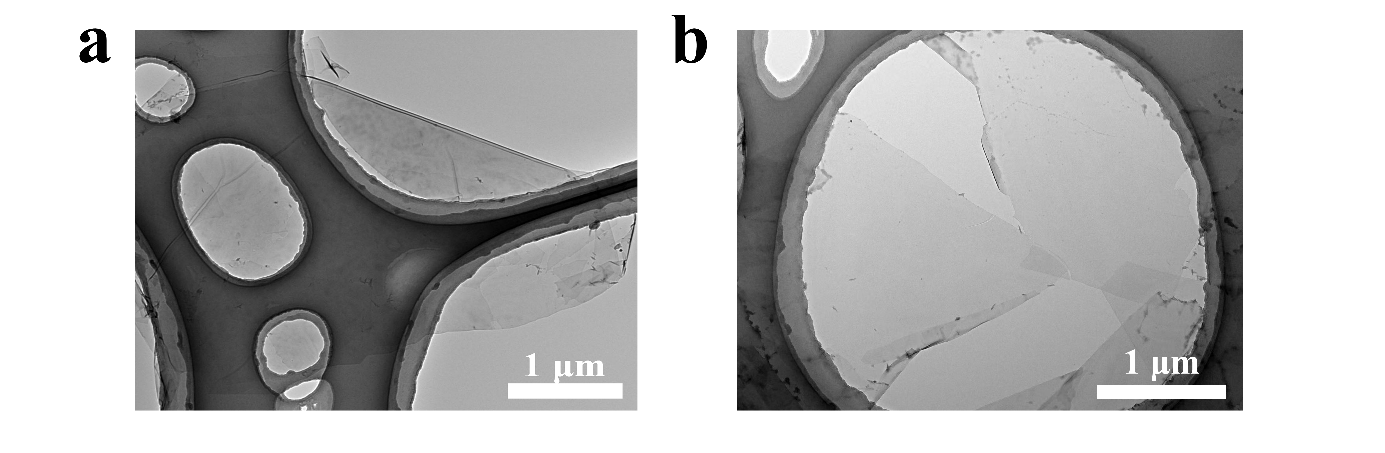


**Fig. S3** TEM images of MXene nanosheets. The size of MXene nanosheets ranges from 1~3 μm


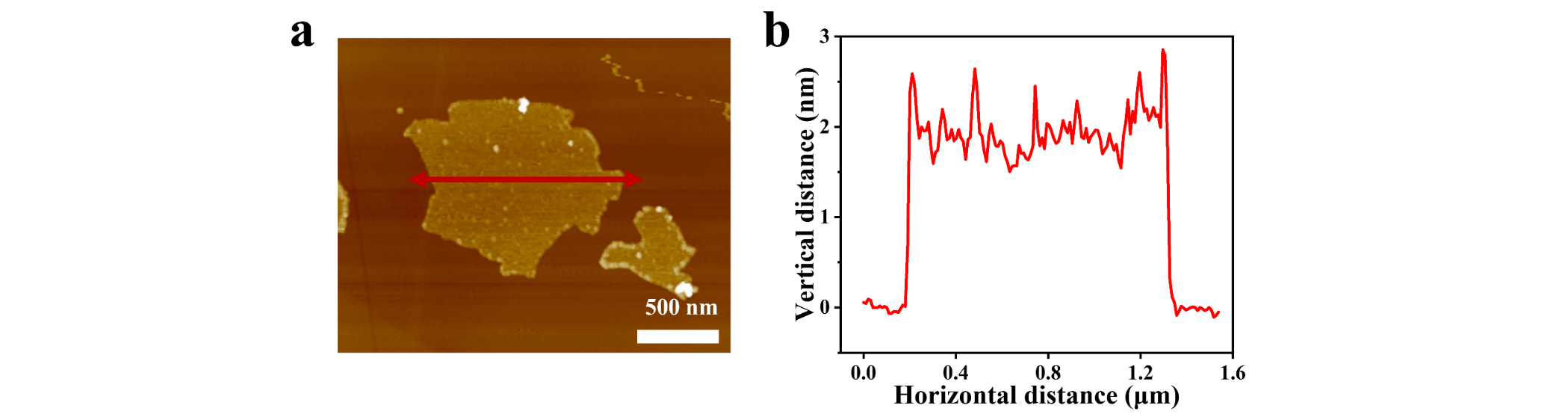


**Fig. S4** Thickness of the MXene nanosheet. (a) AFM image for the MXene nanosheet. (b) Vertical distance for the MXene nanosheet in (a). The thickness of the MXene nanosheet is ~1.9 nm


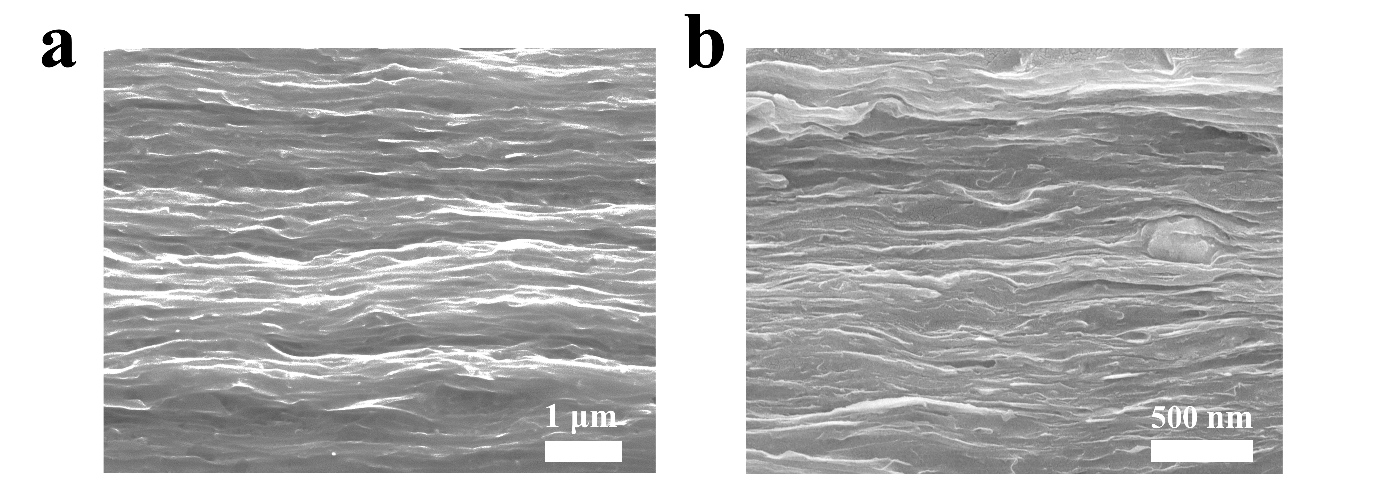


**Fig. S5** SEM images of the intermediate layer in the S-XM film


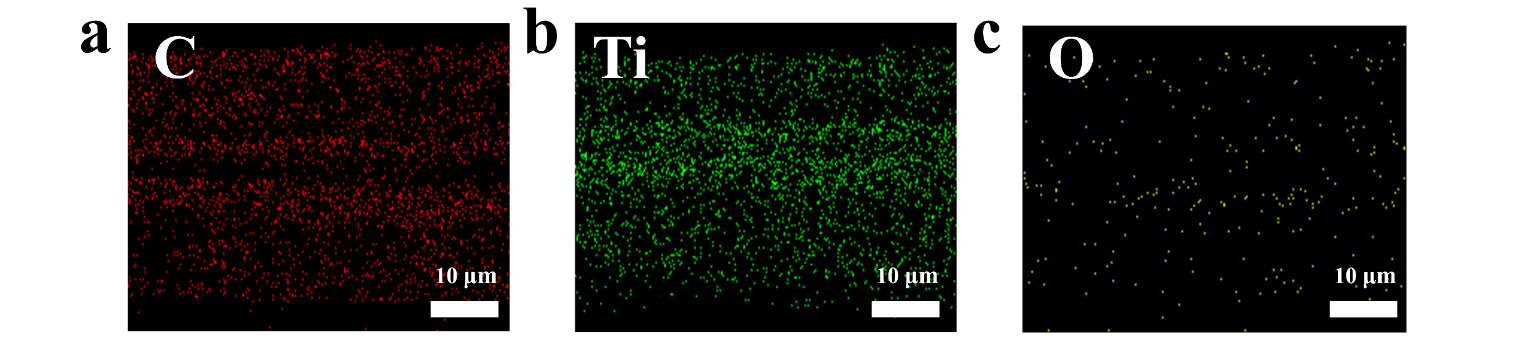


**Fig. S6** The EDS spectra of elemental (**a**) Ti, (**b**) C and (**c**) O in the cross-section of S-XM films


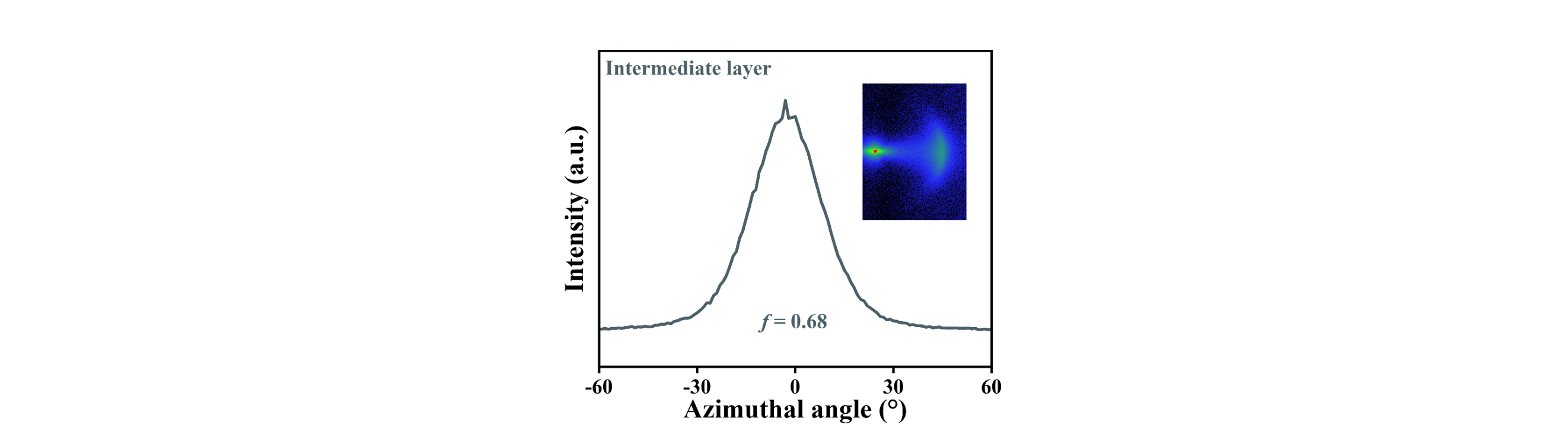


**Fig. S7** WAXS pattern of the isolated intermediate layer in the S-XM film. WAXS patterns for an incident Cu-Kα X-ray beam parallel to the film plane and corresponding azimuthal scan profiles for the (002) peak for films


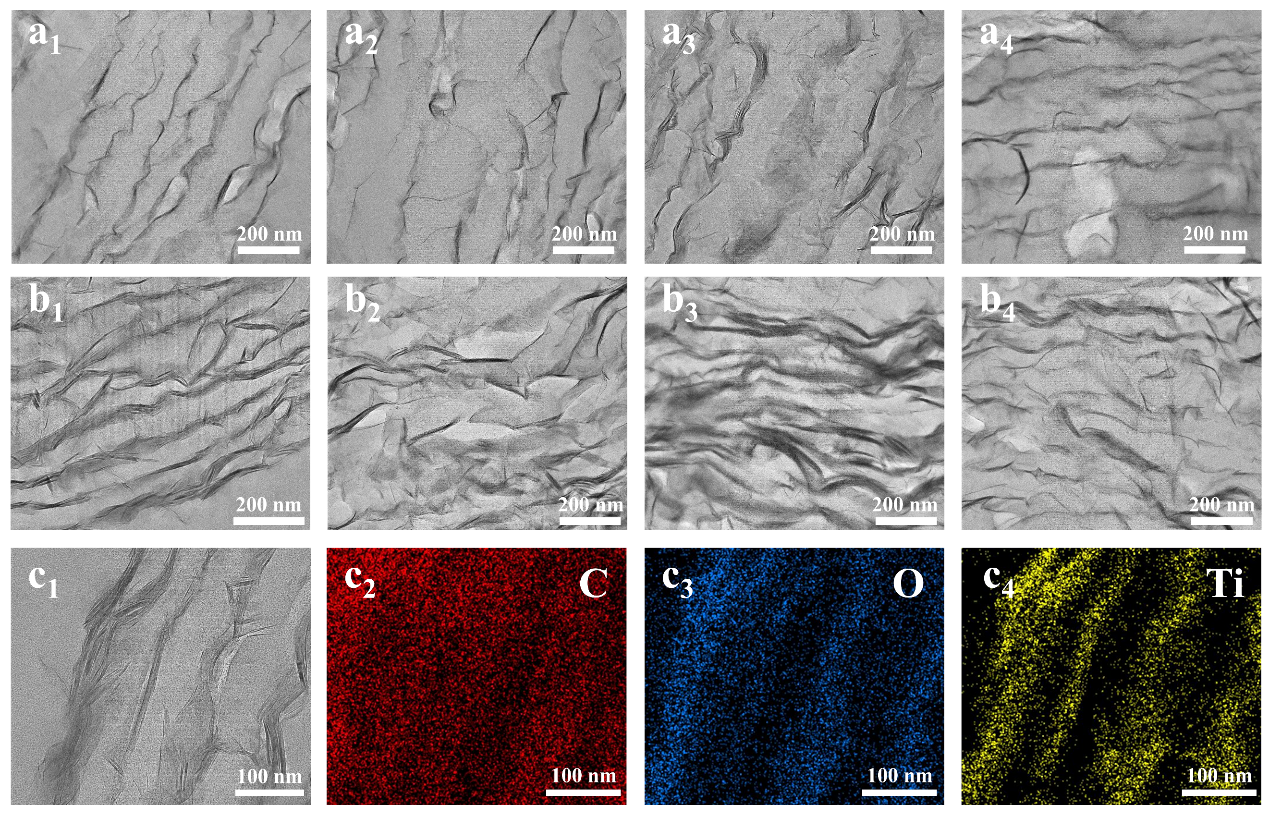


**Fig. S8** The TEM image of the film cross-section and elemental mapping. (a_1_-a_4_) TEM images of the outer layer cross-section of the S-XM film. (b_1_-b_4_) TEM images of the middle layer cross-section of the S-XM film. (c_1_-c_4_) TEM images of the middle layer of the S-XM film at higher magnification, along with corresponding elemental mappings for C, O, and Ti


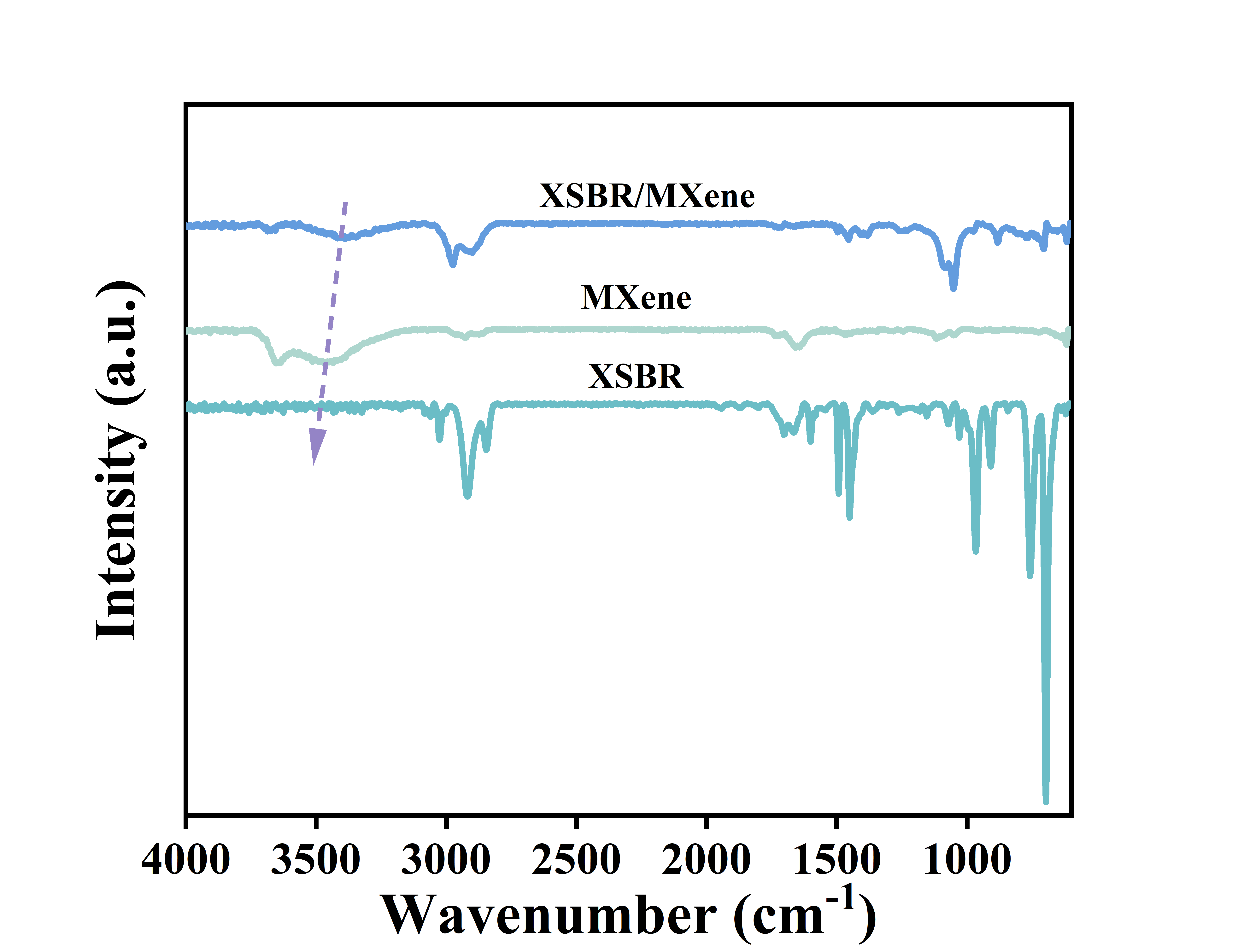


**Fig. S9** FTIR spectra of XSBR films, MXene films, and XM composite films

During the introduction of MXene into XSBR, the -OH vibrational wavenumber of the MXene nanosheets shifted from ~3469 to ~3388 cm^-1^, indicating the formation of hydrogen bonds between MXene and XSBR.


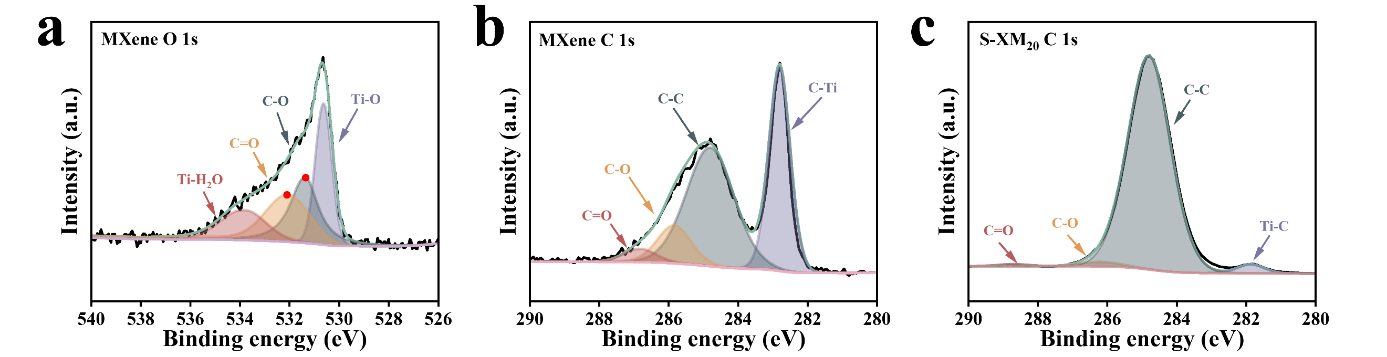


**Fig. S10** XPS spectra of MXene and S-XM_20_ film. (**a**) O 1s XPS spectra of MXene film. (**b**) C 1s XPS spectra of MXene. (**c**) C 1s XPS spectra of S-XM_20_ film

In the O 1s XPS spectrum of MXene, the C–O and C=O peaks are located at 531.35 eV and 532.10 eV, respectively. After introducing XSBR, the C–O and C=O peaks shift to 532.08 eV and 532.98 eV, respectively, indicating that both peaks move to higher binding energies. In the C 1s spectrum, the C–O and C=O peaks shift from 285.84 eV to 286.20 eV and from 286.82 eV to 288.70 eV, respectively. These shifts toward higher binding energies provide further evidence for the successful formation of hydrogen bonds between XSBR and MXene.


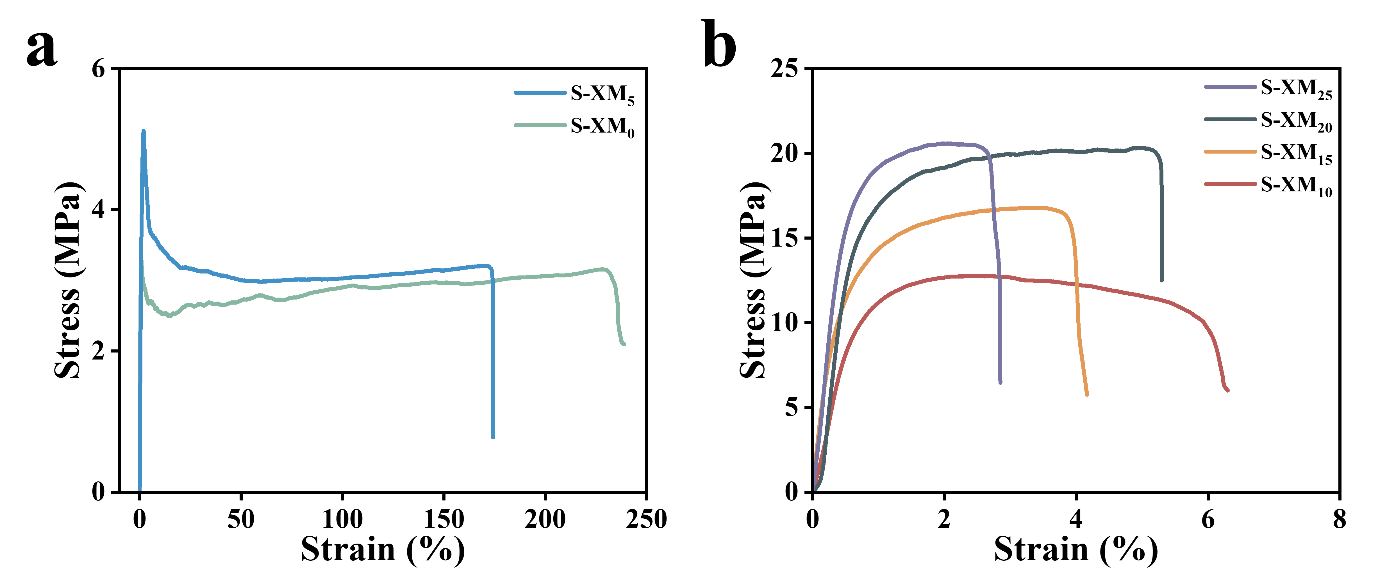


**Fig. S11** Tensile stress–strain curves of S-XM films with varying MXene contents in the outer layer

As the MXene content in the outer layer increased, the tensile strength of the S-XM films significantly improved, rising from 3.37 MPa (S-XM_0_) to 20.58 MPa (S-XM_25_).


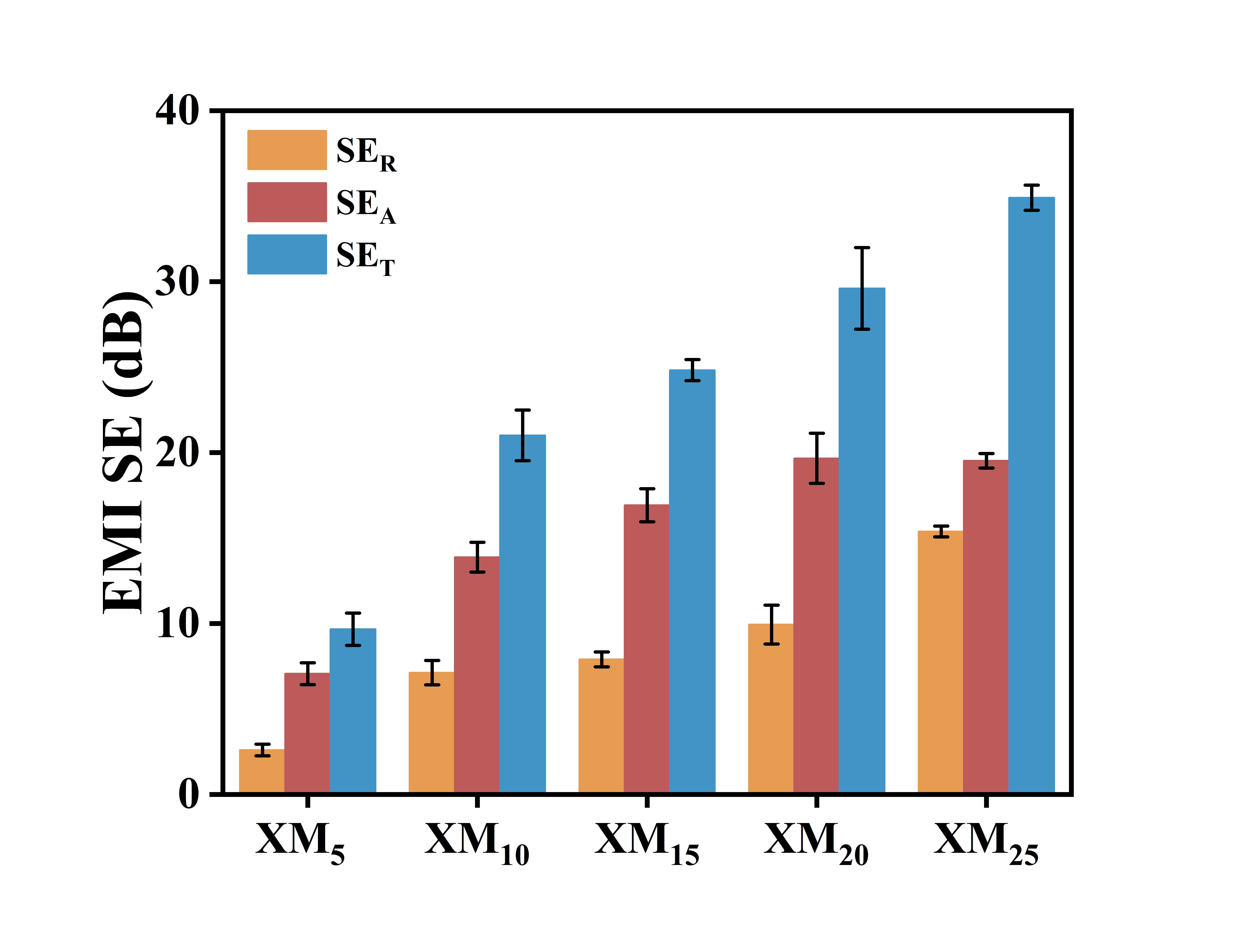


**Fig. S12** SE_R_, SE_A_, and SE_T_ values of single-layer XM films

The absolute shielding effectiveness values of single-layer XM films with varying MXene contents, labeled XM_5_, XM_10_, XM_15_, XM_20_, and XM_25_, range from 9.8 to 35.7 dB. The EMI SE of the XM_15_, XM_20_, and XM_25_ films exceed the commercial standard by 20 dB. Both SE_A_ and SE_R_ significantly contribute to the SE_T_.


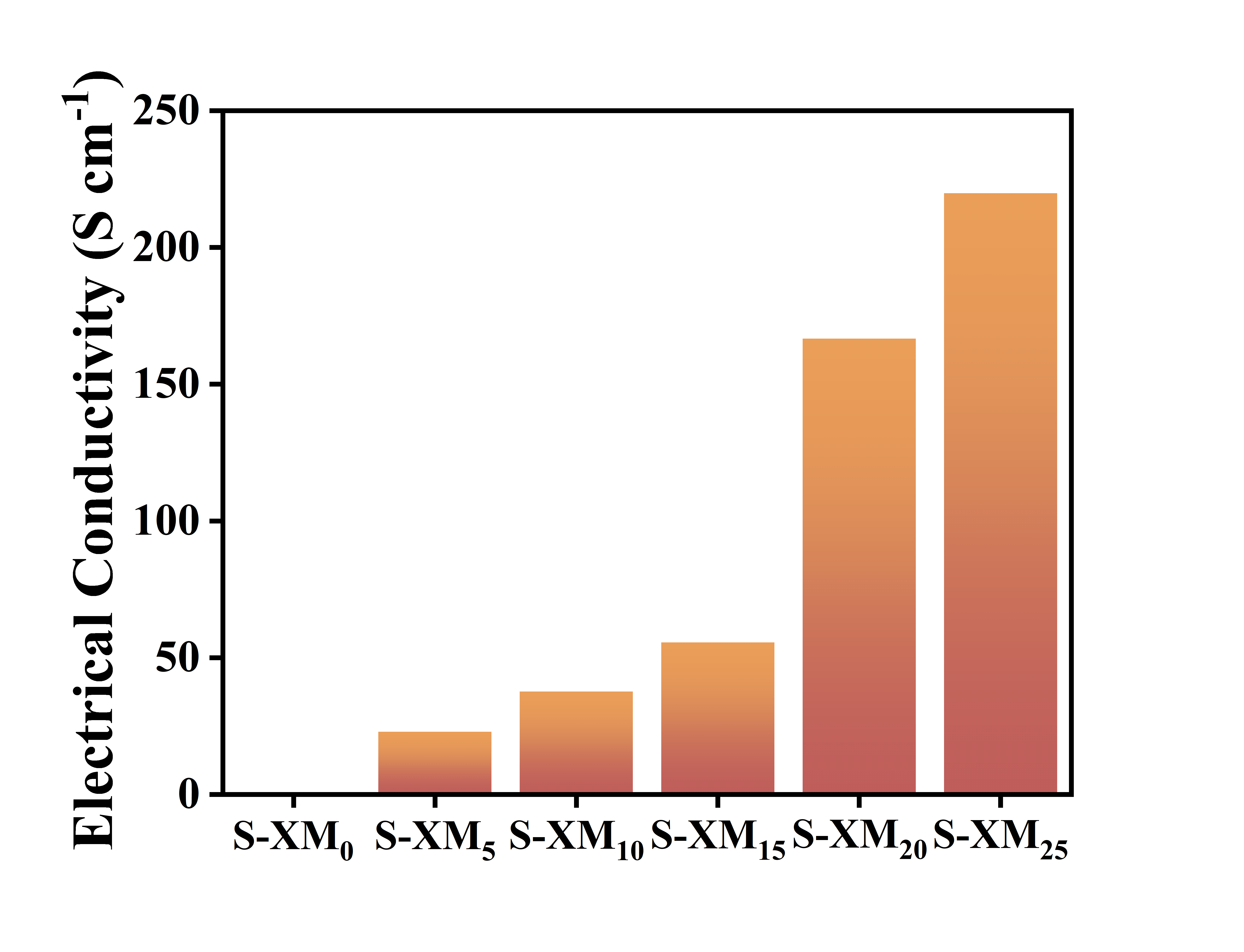


**Fig. S13** Electrical conductivity of S-XM films

Results show that conductivity increases with the MXene content in the outer layers, with S-XM_20_ films achieving a conductivity of 166.7 S cm^-1^. This enhancement is primarily attributed to the formation of a denser and more continuous conductive network within the film, which promotes efficient electron transport and improves overall electrical conductivity.


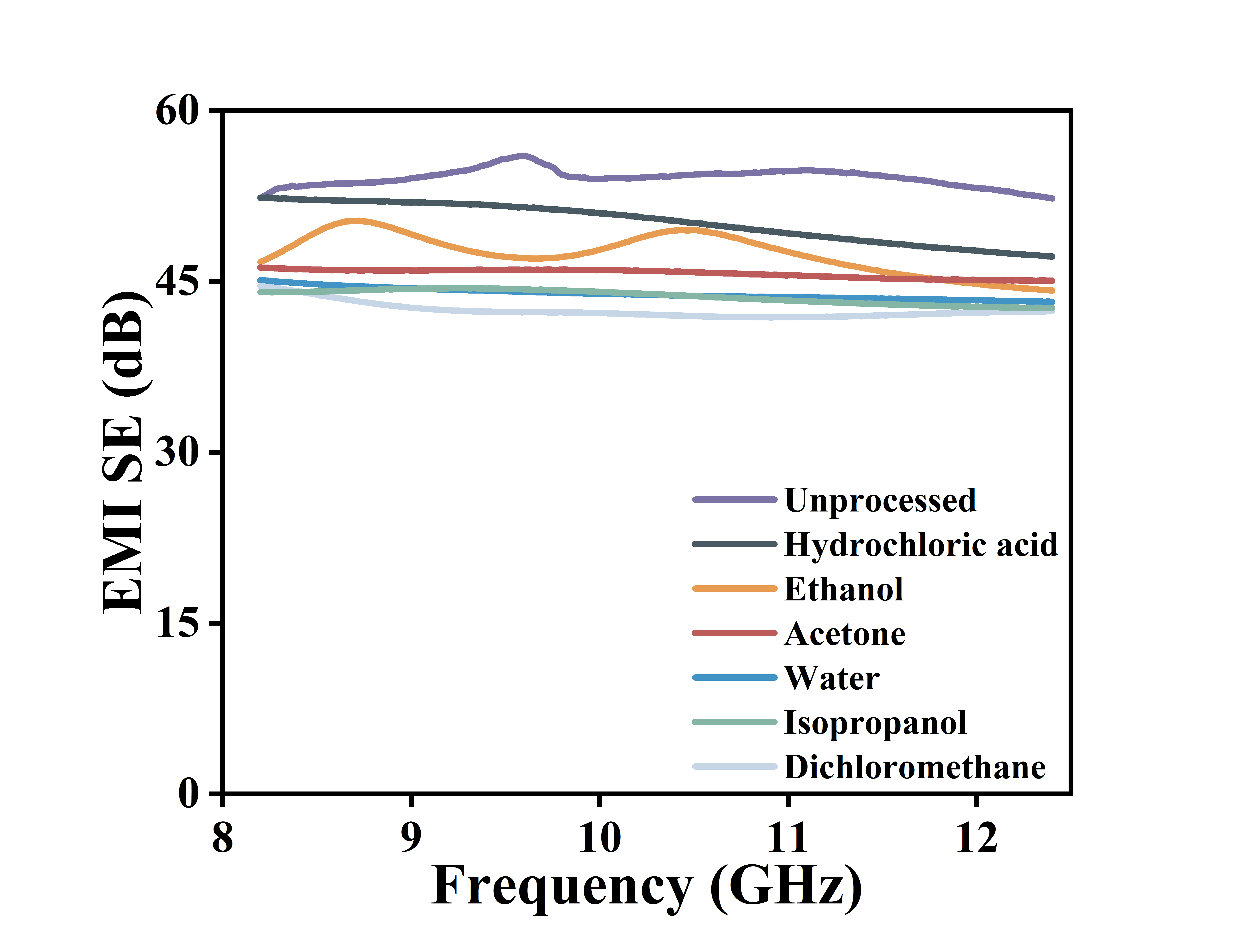


**Fig. S14** Evaluation of the EMI SE of the S-XM_20_ film after 48 h of immersion in different solvents

After 48 h of immersion in different solvents (hydrochloric acid, ethanol, acetone, water, isopropanol, and dichloromethane), the EMI shielding performance of the film decreases slightly, with the EMI SE remaining above 40 dB. This indicates that the film maintains stable EMI shielding properties.


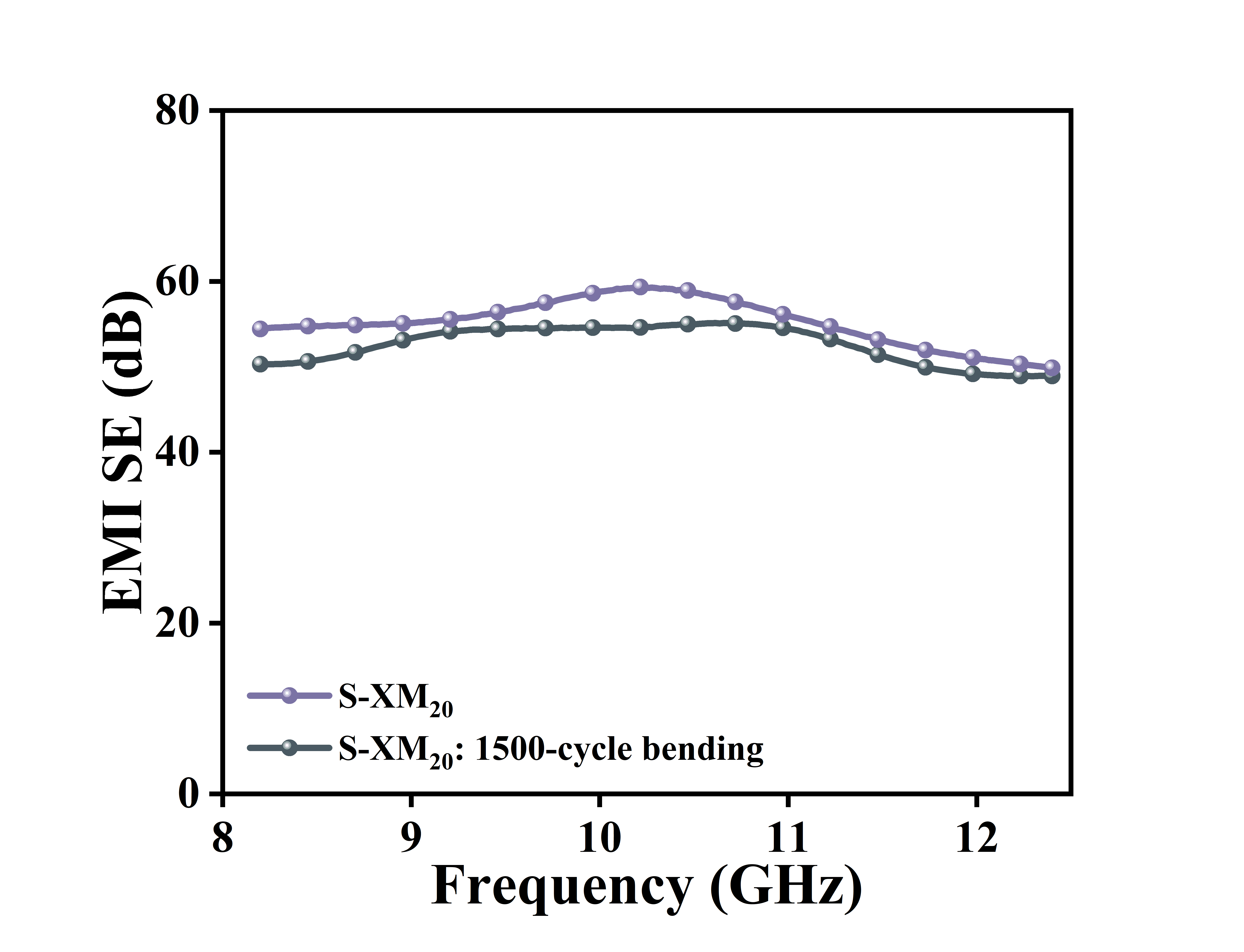


**Fig. S15** Comparison of the EMI SE of the S-XM_20_ film before and after 1,500 bending cycles


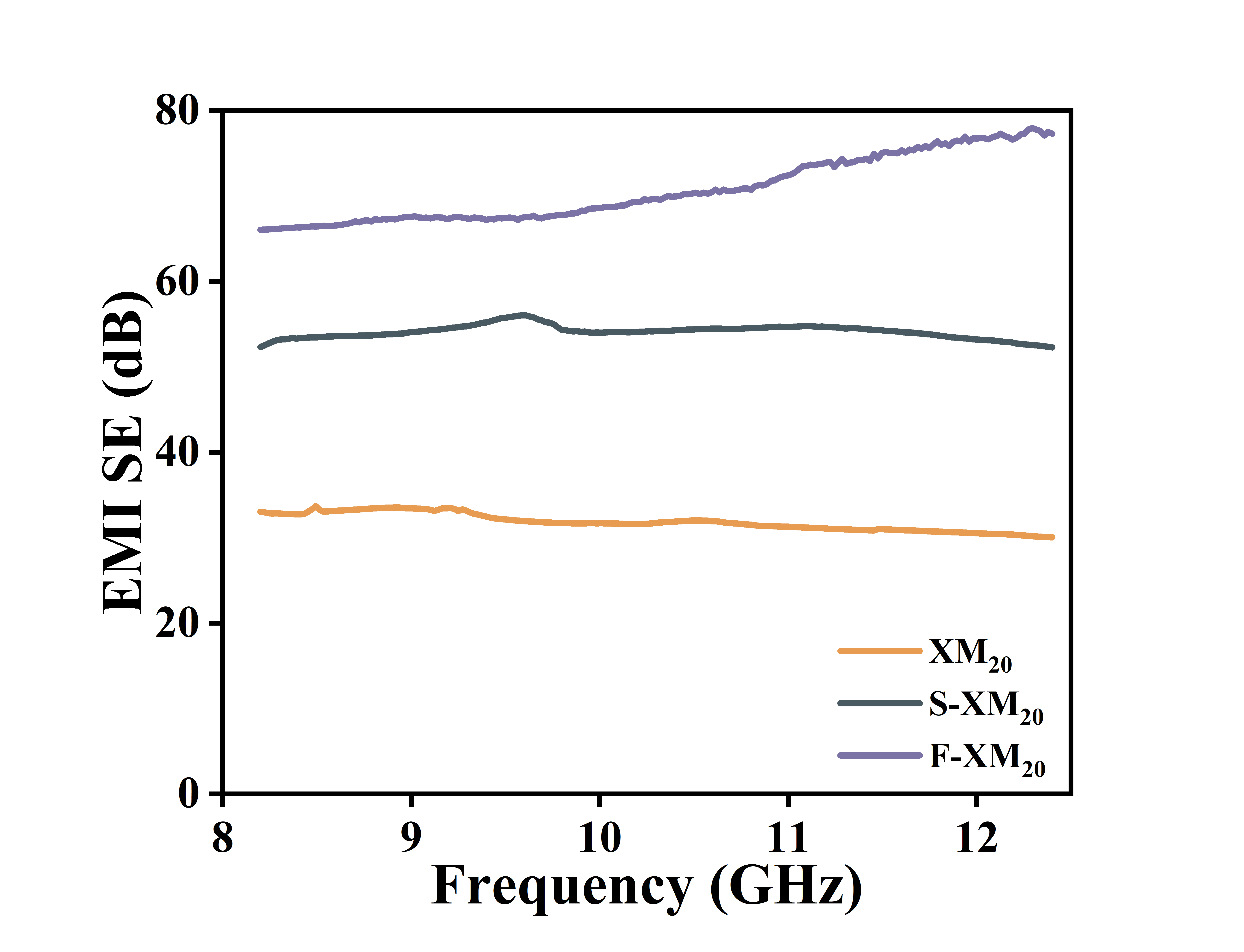


**Fig. S16** Comparison of the EMI SE of single-layer, three-layer, and five-layer XM films

Taking full advantage of the blade coating technology, XM films with different layered structures (single, three, and five layers) were fabricated, and their EMI SE values were compared. The results show that the EMI SE of the F-XM_20_ film, with a thickness of 65 μm, reaches as high as 70 dB, thus confirming the scalability of blade coating technology in the fabrication of layered structures.


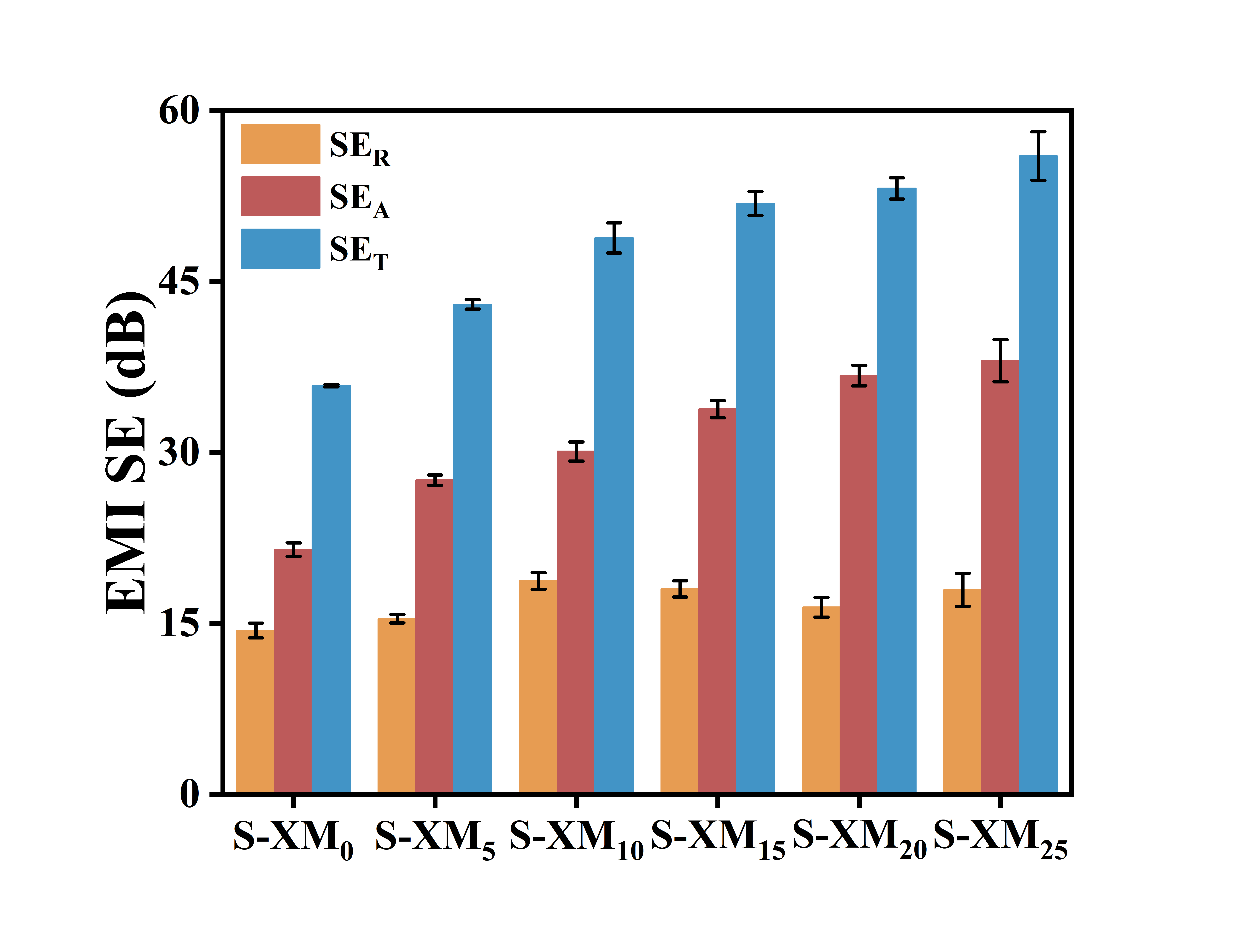


**Fig. S17** SE_R_, SE_A_, and SE_T_ values of S-XM films


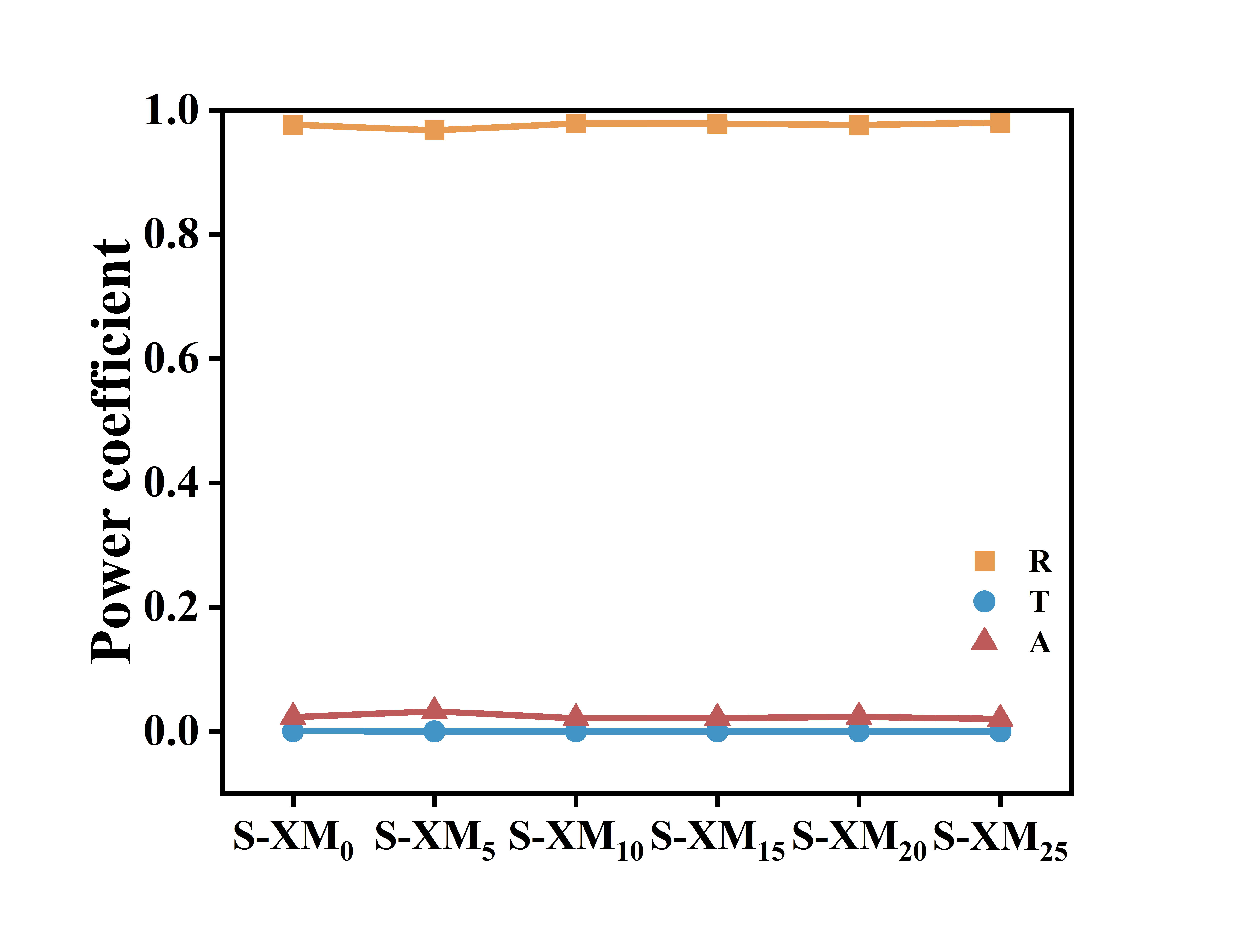


**Fig. S18** Absorption Coefficient (A), Reflection Coefficient (R), and Transmission Coefficient (T) of the S-XM Films


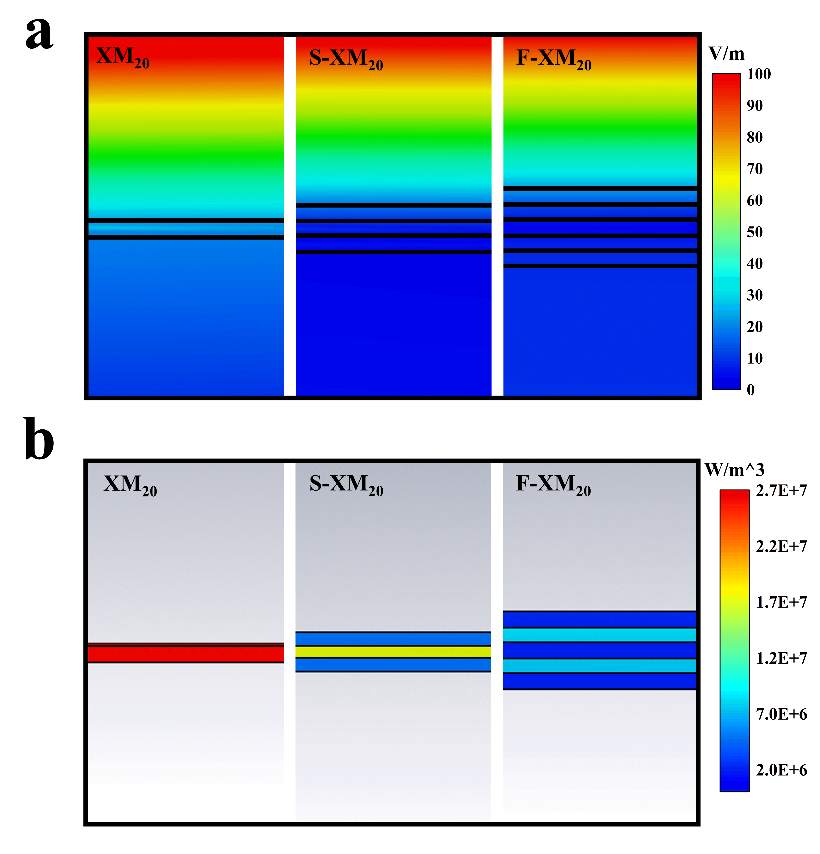


**Fig. S19** The finite element simulation of films without layered structures. (**a**) Electric field strength (**b**) Energy loss

**
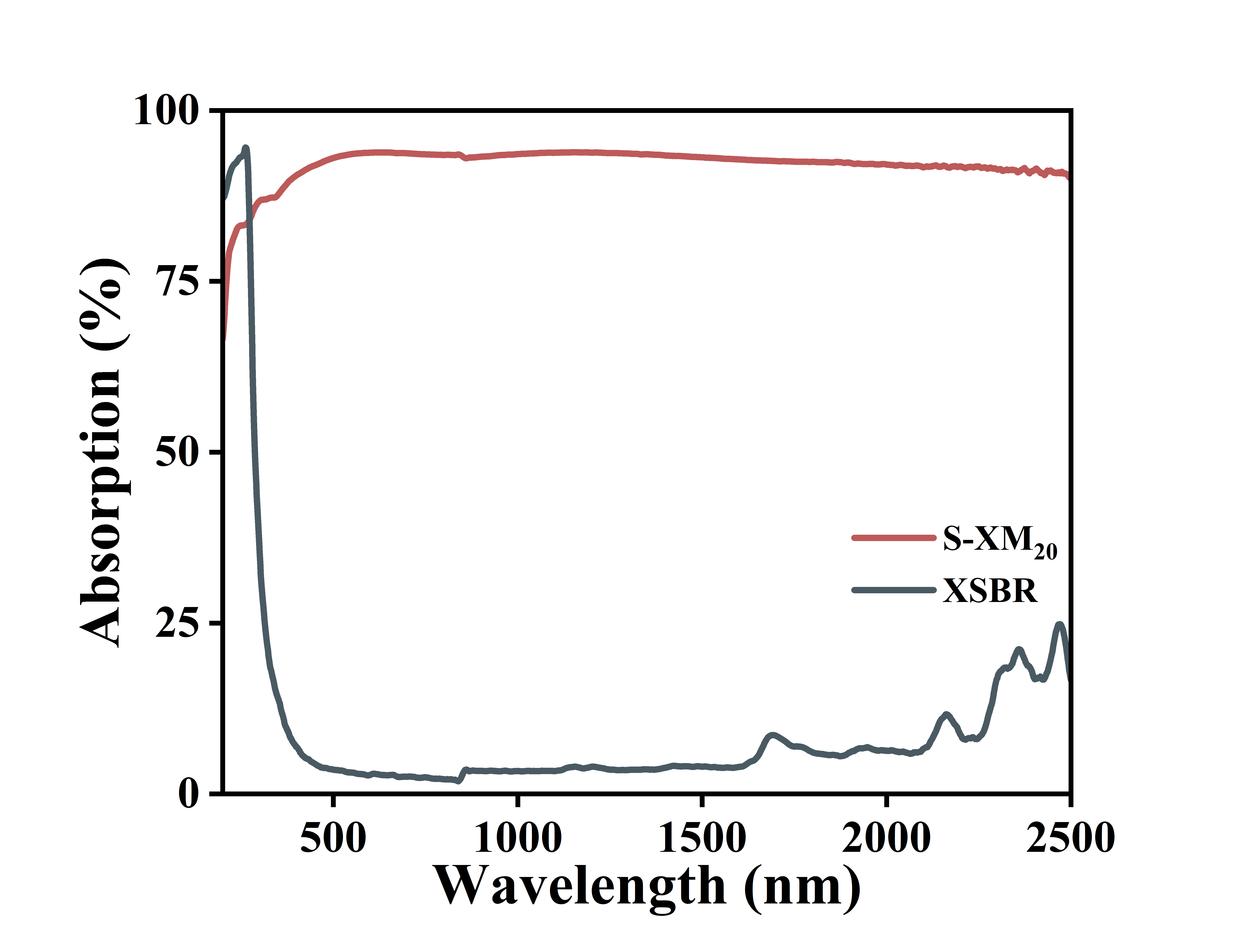
**

**Fig. S20** UV–Vis–NIR absorption spectra of XSBR and S-XM_20_ films


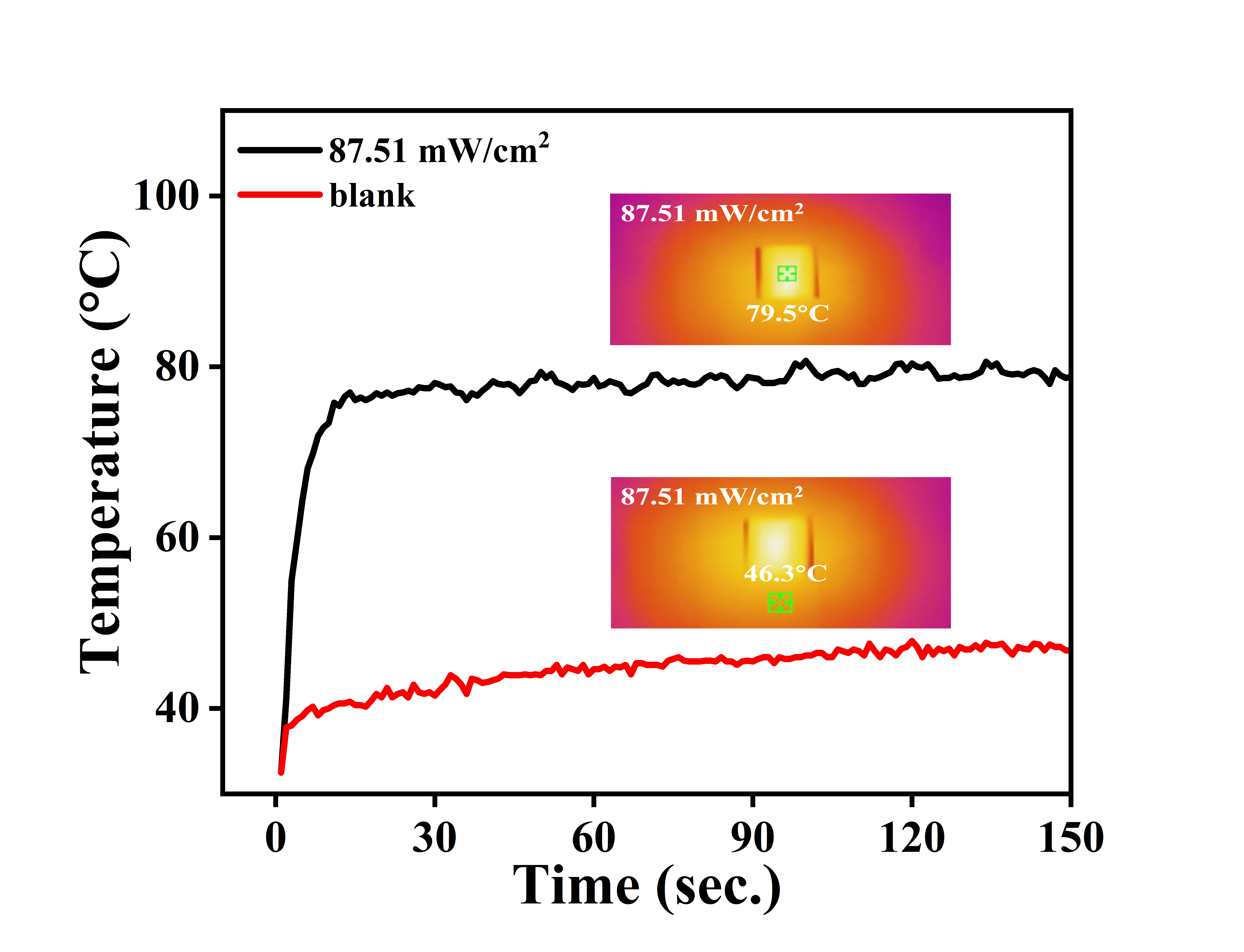


**Fig. S21** Temperature variation curves and corresponding steady-state infrared thermographic images of the S-XM film and substrate at a radiation intensity of 87.51 mW/cm^2^

By comparing the temperature changes of the film and the background plate under the same radiation intensity, it is demonstrated that the background plate has a negligible effect on the photothermal properties of the film.





**Fig. S22** Temperature curve of the S-XM_20_ film over 150 switching cycles, with radiation intensity varying from 0 to 45.51 mW/cm^2^


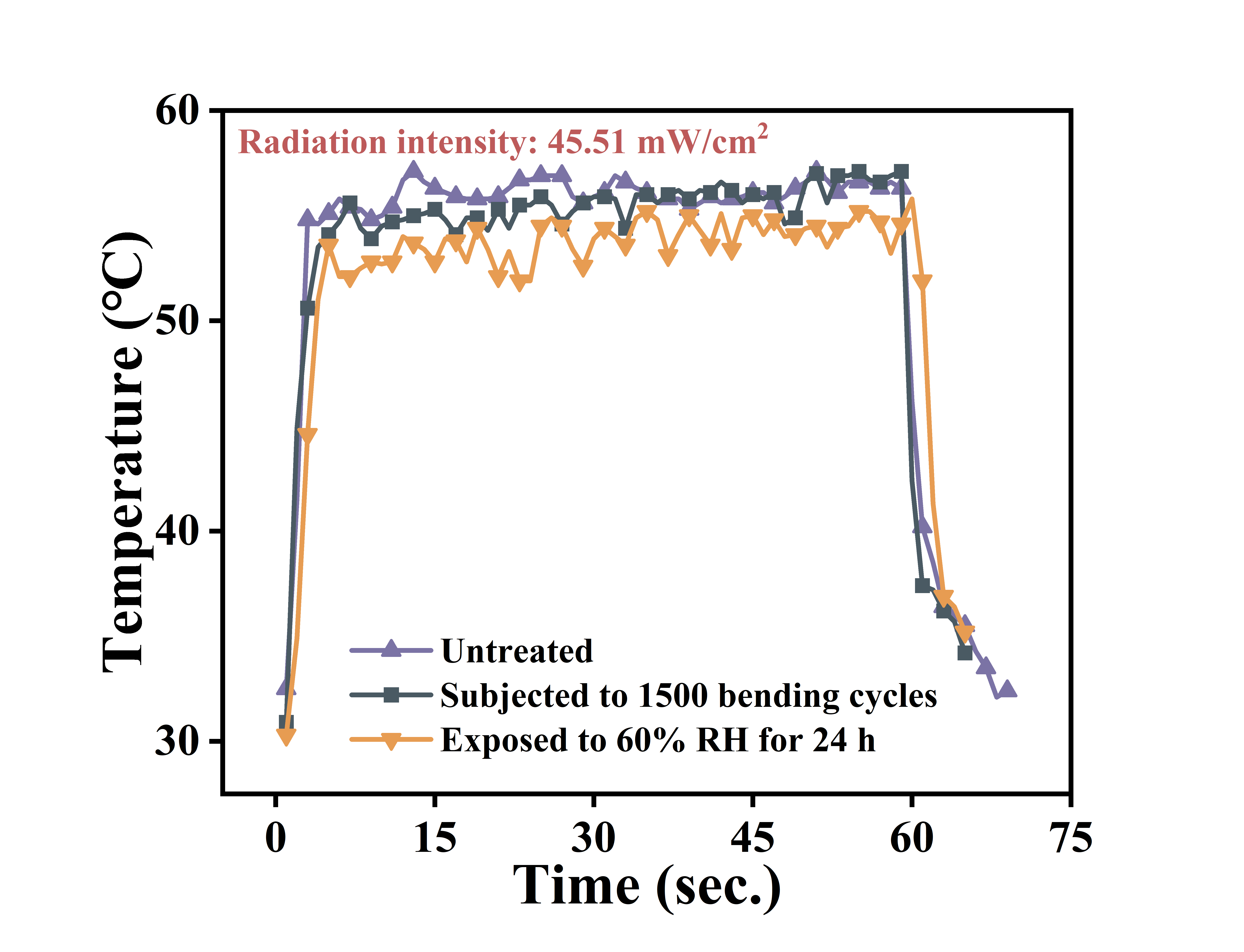


**Fig. S23** Comparison of the photothermal performance of the untreated film, the film treated with simulated human skin humidity, and the film subjected to repeated mechanical deformation


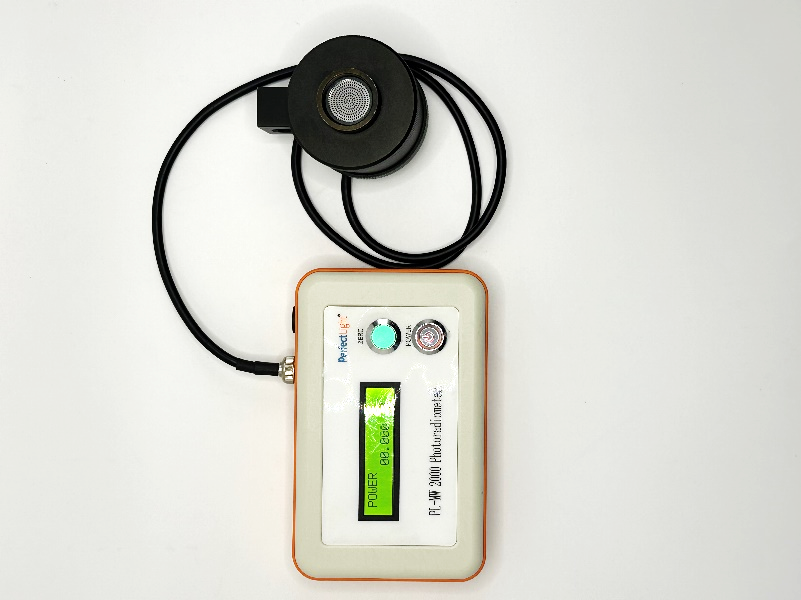


**Fig. S2****4** Schematic diagram of an optical power mete


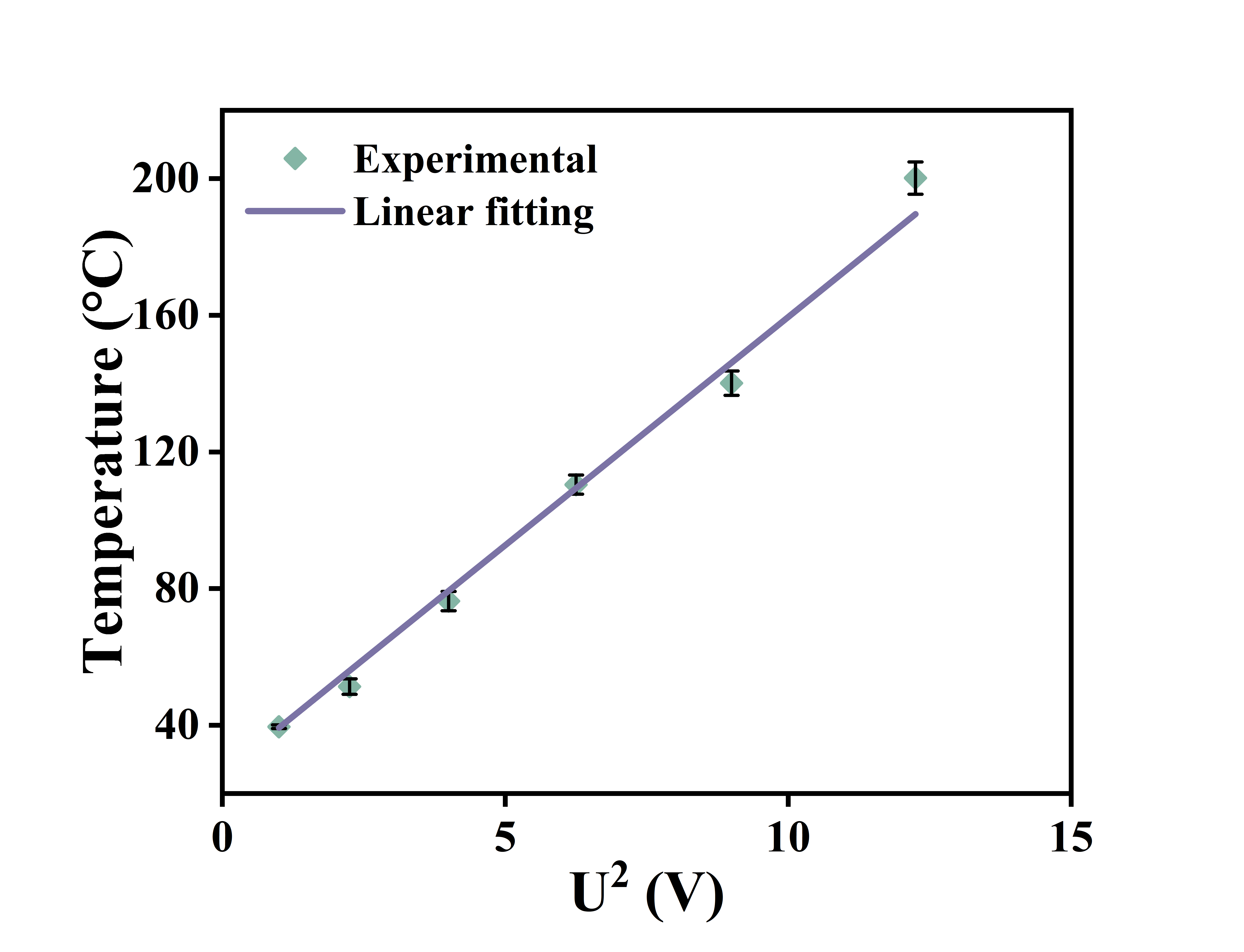


**Fig. S25** Linear fit of the steady-state Joule heating temperature of the S-XM_20_ film versus the square of the applied voltage

**
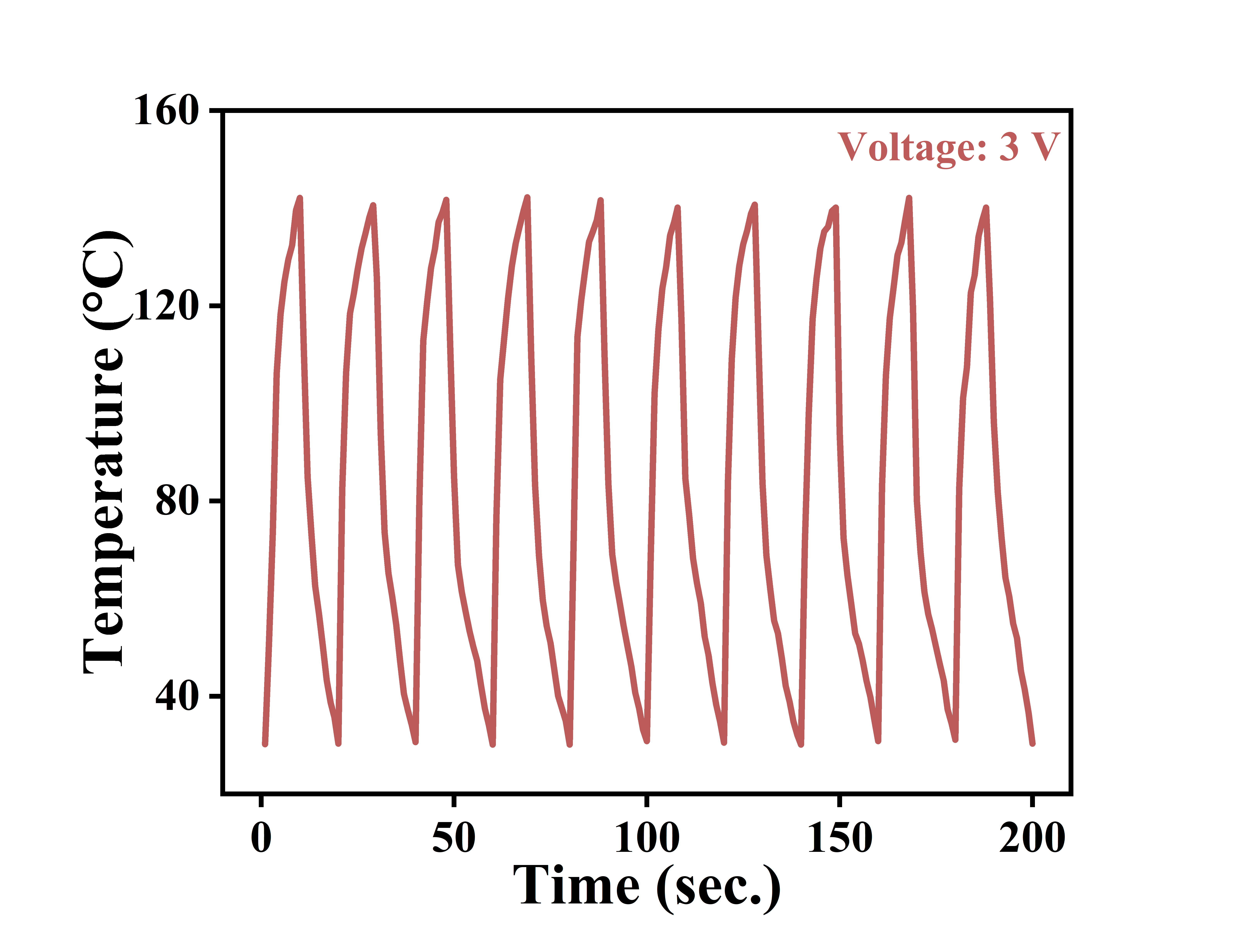
**

**Fig. S26** Temperature curve of the S-XM_20_ film over 10 switching cycles (with the driving voltage varying from 0V to 3V)


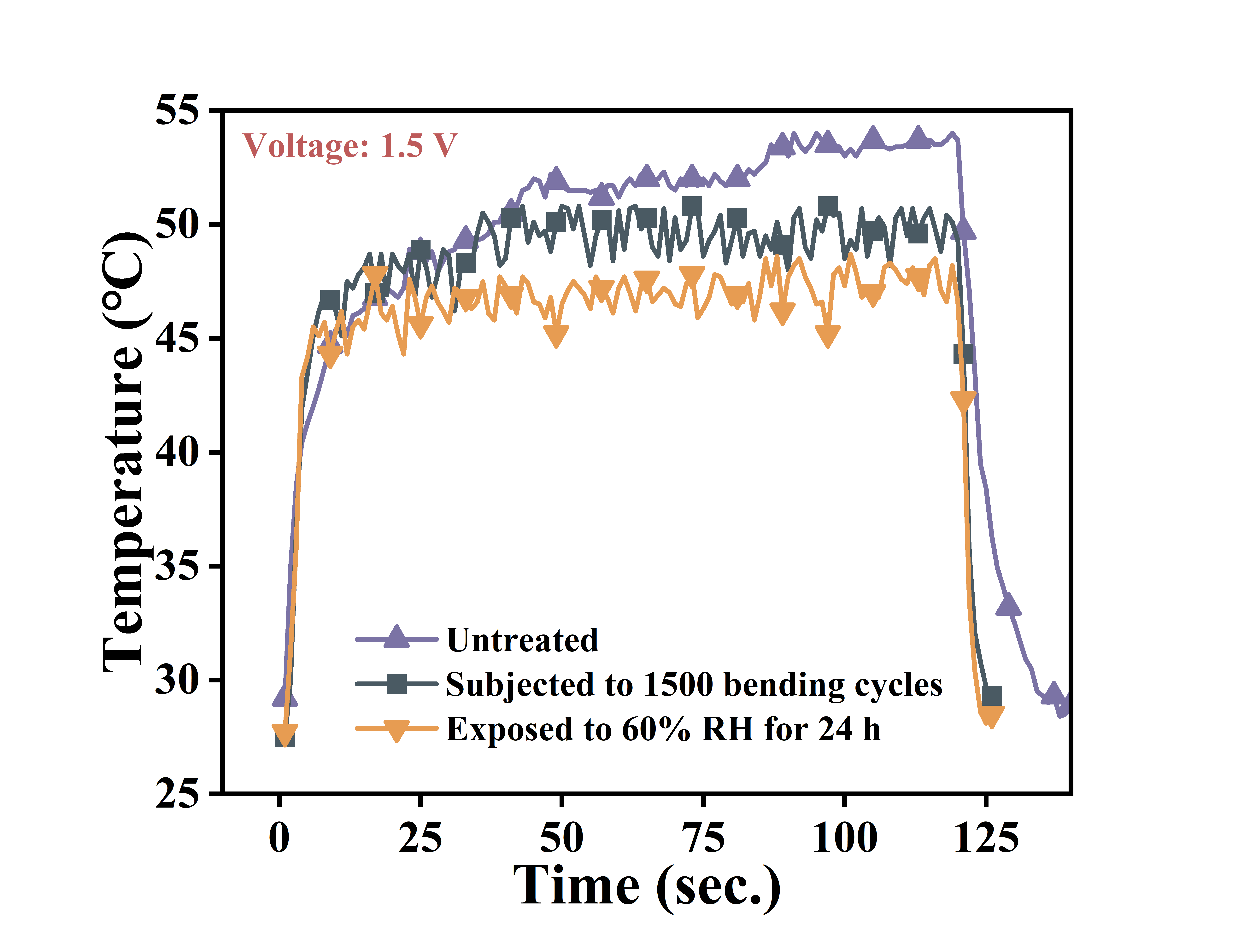


**Fig. S27** Comparison of the Joule heating performance of the untreated film, the film treated with simulated human skin humidity, and the film subjected to repeated mechanical deformation


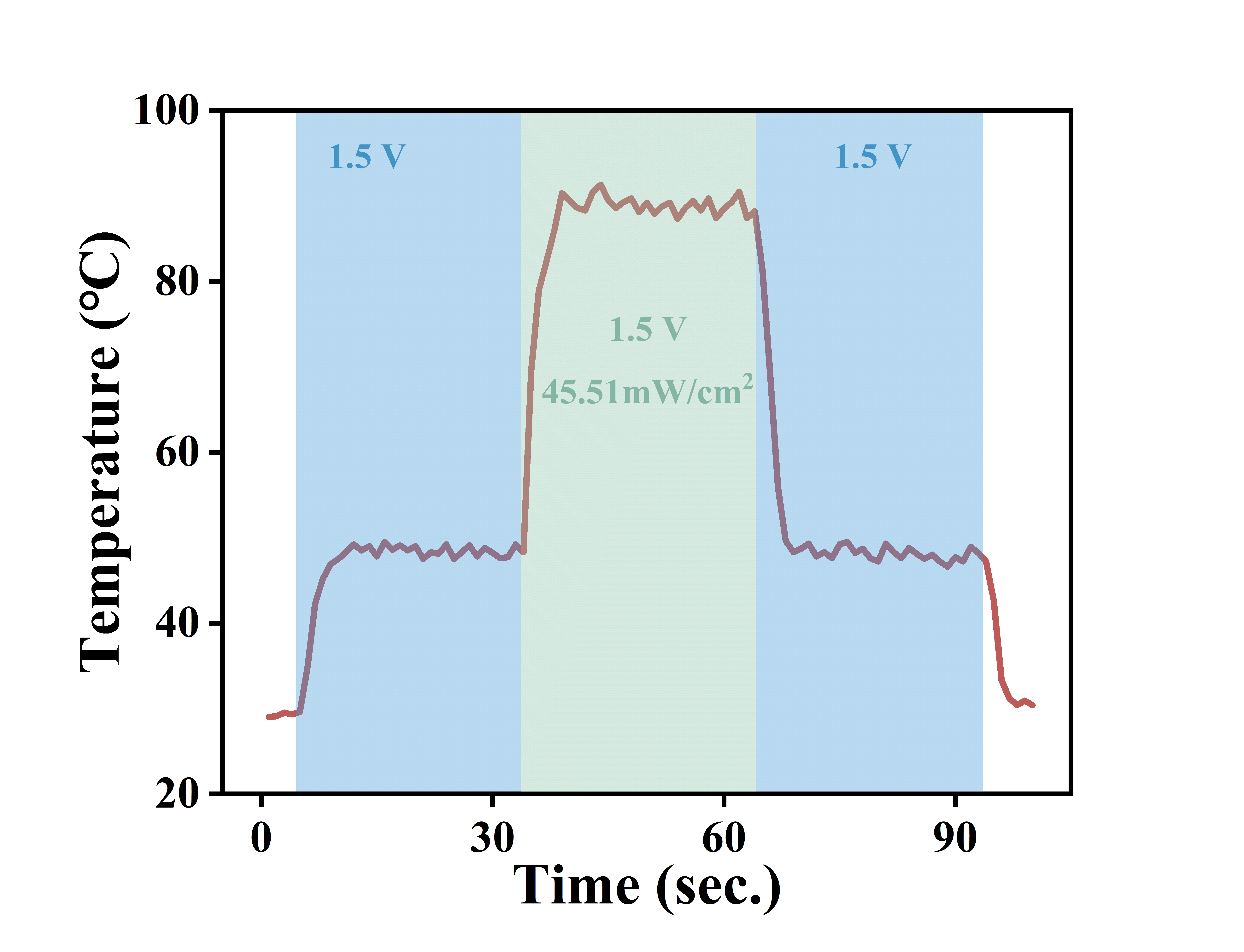


**Fig. S28** Joule heating and photothermal synergistic heating curve


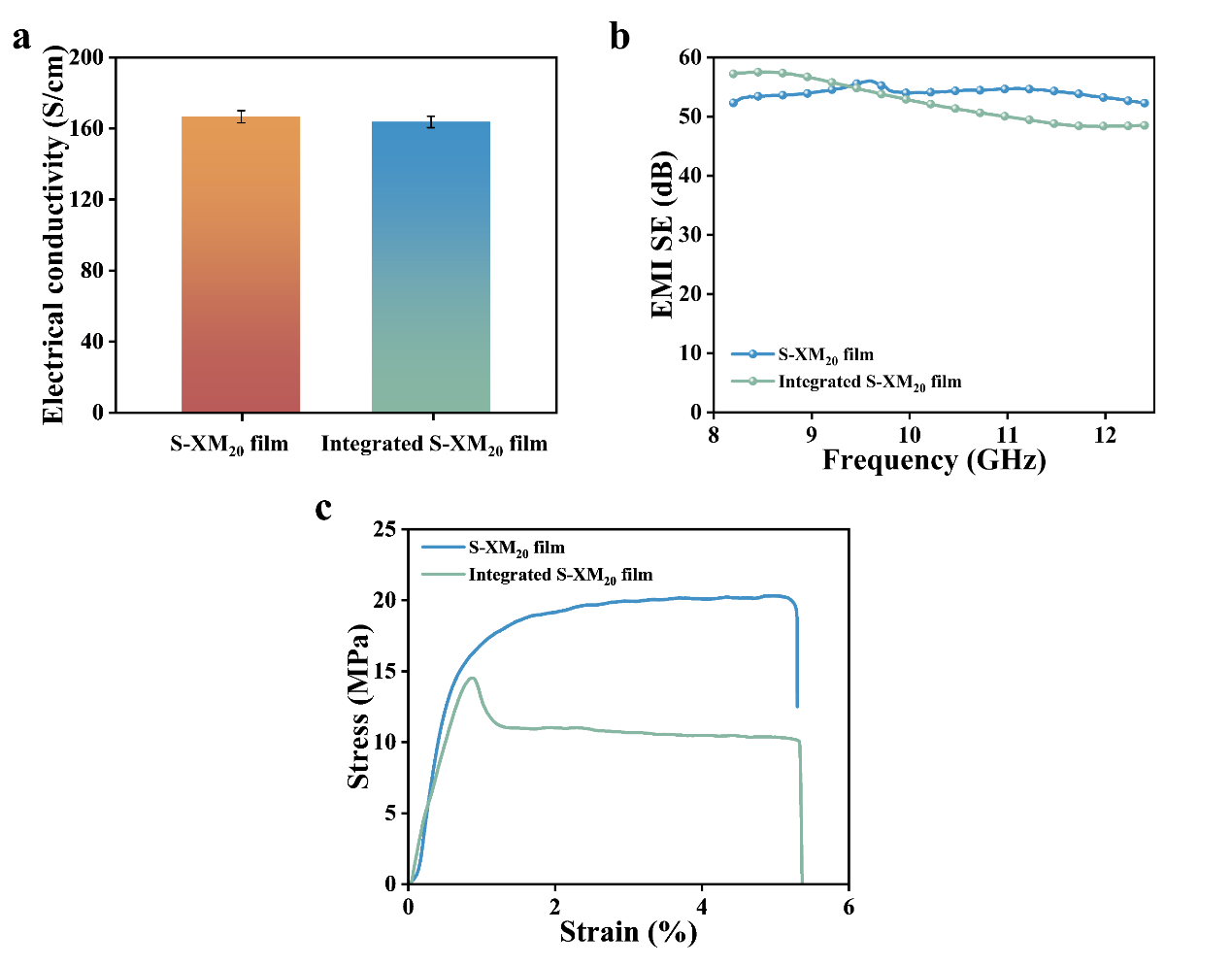


**Fig. S29** Changes in the (**a**) electrical conductivity, (**b**) EMI shielding performance, and (**c**) mechanical properties of the S-XM_20_ film before and after encapsulation


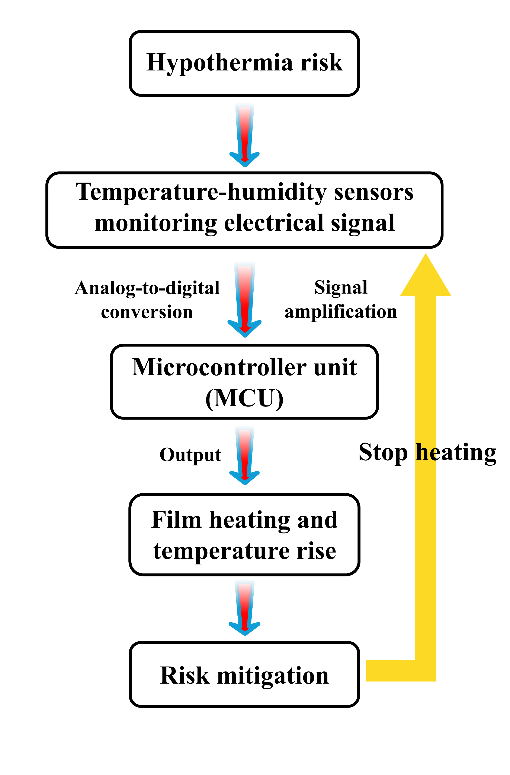


**Fig. S30** The operational flowchart of the intelligent thermal management system


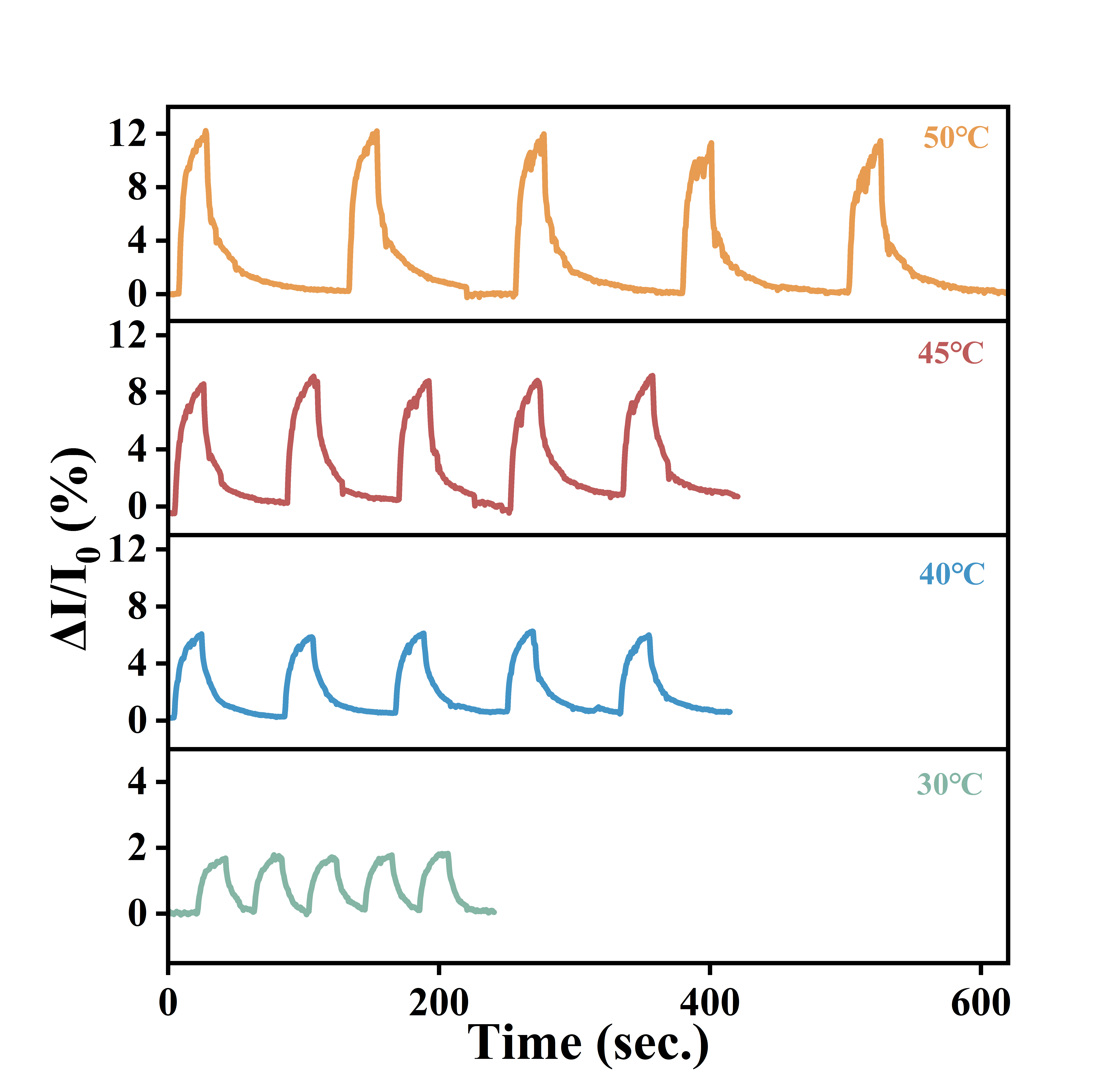


**Fig. S****31** Cyclic stability tests of the temperature sensor within the human comfort temperature range (30°C~50°C)

Electrical signal responses at different temperatures show that *ΔI/I₀* remains stable with high reproducibility over five cycles.


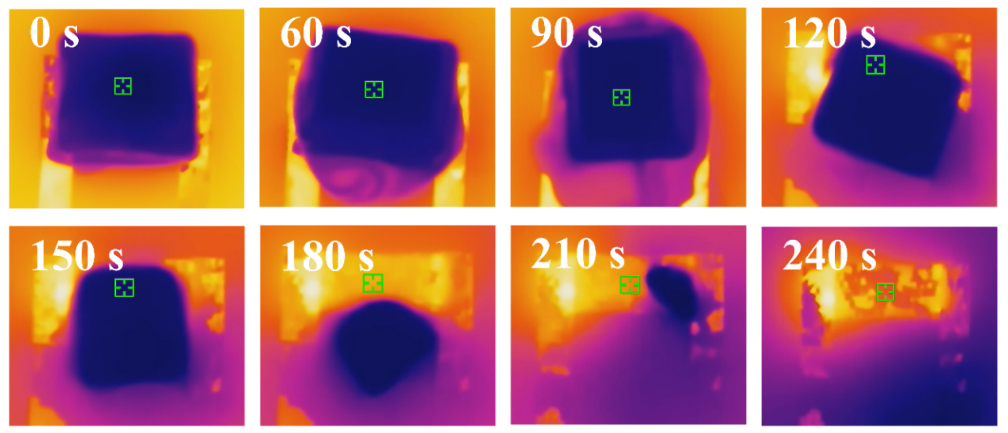


**Fig. S32** Infrared thermal images of the S-XM film illustrating the synergistic de-icing process through personal thermal management and temperature sensing

To simulate ice formation under extreme environmental conditions, ice was placed on the surface of the film. Upon detecting a rapid temperature drop, the temperature sensor promptly generated an electrical signal and transmitted it to the film’s thermal management system. The system then activated Joule heating to increase the film’s temperature, with the complete de-icing process captured by infrared thermal imaging.

**
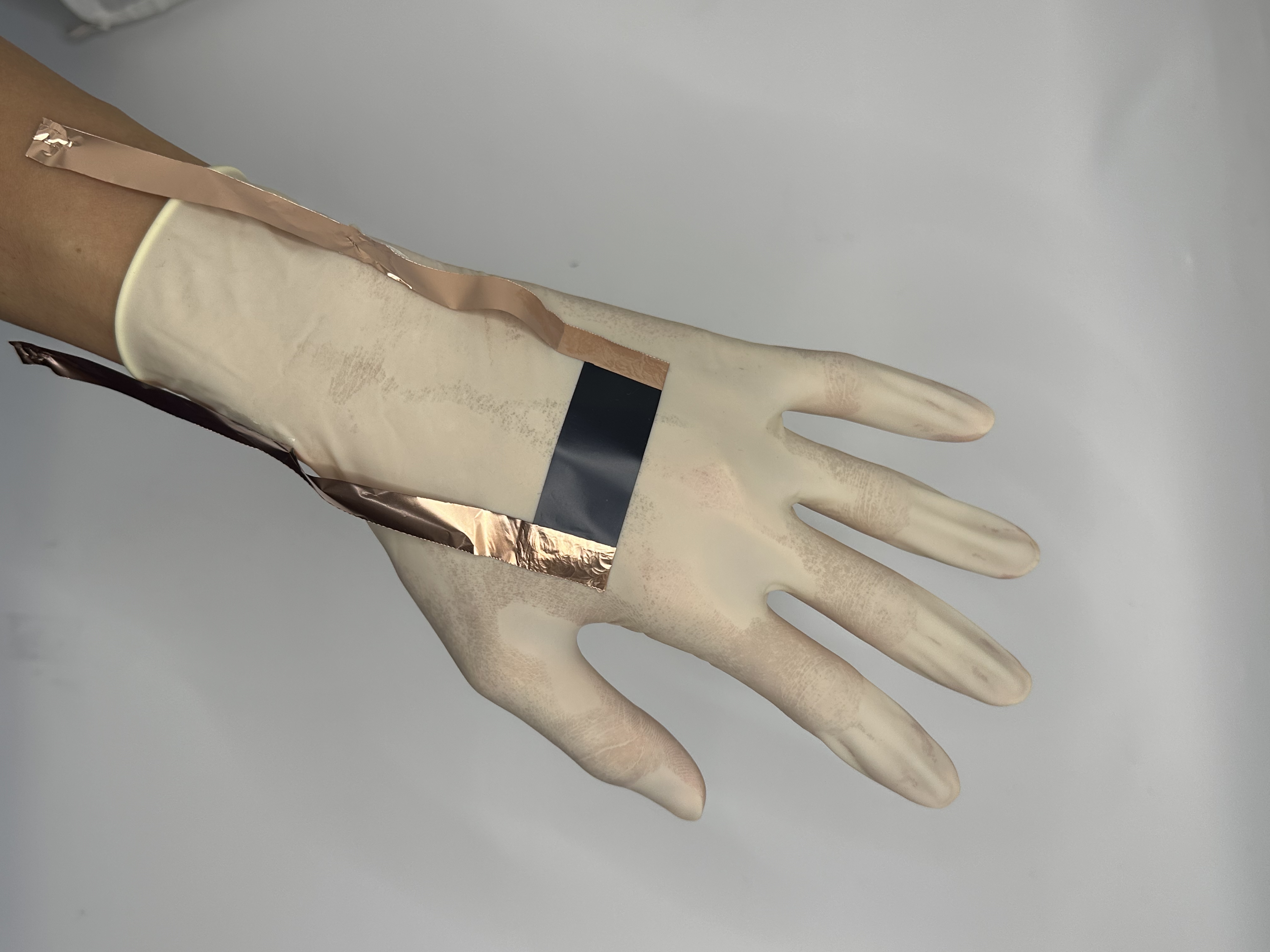
**

**Fig. S33** Schematic diagram of wearable thermal therapy based on the Joule heating performance of the film

**Table S2** Comparison of EMI shielding performance data

|  | Materials | Thickness (mm) | EMI SE (dB) | SE/t (dB mm^-1^) | Refs. |
| --- | --- | --- | --- | --- | --- |
|  | **rubber-based films** |  |  |  |  |
| 1 | RGO/ZnO-XNBR | 1 | 34.2 | 34.2 | [S1] |
| 2 | Fe_3_O_4_@CNT/NR | 2 | 63.8 | 31.9 | [S2] |
| 3 | PCB/EPDM | 1 | 49.94 | 49.94 | [S3] |
| 4 | CNT/SiO_2_/PDMS | 2 | 52.2 | 26.1 | [S4] |
| **5** | MXene/NR | 0.251 | 53.6 | 213.55 | [S5] |
| 6 | CF@Fe_2_O_3_/(BN/SR) | 0.28 | 37.7 | 134.64 | [S6] |
| 7 | F@rLG-Rx/CNRy | 2 | 34.4 | 17.2 | [S7] |
| 8 | MXene/Fe_3_O_4_/SR | 2.2 | 55.5 | 25.23 | [S8] |
| 9 | CI@SiO_2_/NA/SR | 1.6 | 64.5 | 40.31 | [S9] |
| 10 | EMA-XNBR-VXC | 1 | 33.5 | 33.5 | [S10] |
| 11 | CNT/hBN/NR | 1.4 | 32.52 | 23.23 | [S11] |
| 12 | MXene/NR | 0.065 | 47.8 | 735.38 | [S12] |
| 13 | MWCNT/PP/EPDM | 1 | 29.8 | 29.8 | [S13] |
| 14 | Ag@s-BN/SR | 2 | 20.9 | 10.45 | [S14] |
| 15 | Fe_3_O_4_@MXene/DOPAC/ENR | 1.2 | 58 | 48.33 | [S15] |
| 16 | Ni@CF/SR | 0.7 | 42.6 | 60.86 | [S16] |
| 17 | MWCNTs/XNBR | 1 | 27.3 | 27.3 | [S17] |
| 18 | PEDOT:PSS/Li-TFSI/XSB | 0.2 | 50 | 250 | [S18] |
| 19 | MXene/NR | 0.065 | 63.5 | 976.92 | [S19] |
| 20 | MXene/WPU/NR | 0.339 | 76.1 | 224.48 | [S20] |
| 21 | Fe_3_O_4_@rGO/NR | 1.8 | 42.4 | 23.56 | [S21] |
| 22 | K-CB/PVA/NR | 0.2 | 37.7 | 188.5 | [S22] |
| 23 | Microcoils/SR | 0.4 | 26.2 | 65.5 | [S23] |
| 24 | GO/SR | 1.7 | 30.42 | 17.89 | [S24] |
| 25 | LM/PDA/SR | 0.1 | 55.1 | 551 | [S25] |
| 26 | CNT@PDA/MXene/XSBR | 0.235 | 58.5 | 248.9 | [S26] |
| 27 | AgNW/MXene/NiCo-C/SR | 1.1 | 89.12 | 81.02 | [S27] |
| 28 | MXene/NR | 0.05 | 34 | 680 | [S28] |
| 29 | PEDOT:PSS/XNBR | 0.076 | 26.8 | 352.6 | [S29] |
| 30 | Fe_3_O_4_@CNT/NR | 0.5 | 26.6 | 531 | [S30] |
|  | **MXene-based films** |  |  |  |  |
| 1 | Zn-MXene/Nonwoven | 0.400 | 57.50 | 143.75 | [S31] |
| 2 | PPy/MXene/PET | 1.300 | 90.00 | 69.23 | [S32] |
| 3 | MXene/Fe-GaInSn/SBS | 0.800 | 63.50 | 79.38 | [S33] |
| 4 | MXene/AgNWs/Nonwoven | 0.600 | 49.70 | 82.83 | [S34] |
| 5 | AgNW@MXene/Wood | 1.000 | 44.96 | 44.96 | [S35] |
| 6 | MXene/ANF | 0.037 | 48.00 | 1297.3 | [S36] |
| 7 | Fe_3_O_4_/MXene/PI | 0.075 | 85.00 | 1133.33 | [S37] |
| 8 | AgNPs@MXene/GF | 1.000 | 52.00 | 52 | [S38] |
| 9 | CNF/PEDOT:PSS/MXene | 0.058 | 76.99 | 1327.41 | [S39] |
| 10 | MXene/GO@MC | 0.185 | 84 | 454.1 | [S40] |
| 11 | MXene/CF | 0.321 | 46.26 | 144.1 | [S41] |
| 12 | BC/MXene/HFO | 0.35 | 67.6 | 193.1 | [S42] |
| 13 | MXene/CNF | 0.105 | 44.5 | 423.8 | [S43] |
| 14 | MXene/BC | 0.116 | 43.7 | 376.7 | [S44] |
| 15 | MXene/TOCNF | 0.038 | 39.6 | 1042.1 | [S45] |
|  | **Sandwich structure films** |  |  |  |  |
| 1 | CuS/PI/PTFE | 0.08 | 57.1 | 713.75 | [S46] |
| 2 | MXene/AgNW/PI | 0.15 | 79.54 | 530.27 | [S47] |
| 3 | SiO_2_@MXene/PDMS | 0.2 | 43.3 | 216.5 | [S48] |
| 4 | MXene/AgNW/CNF/PDMS | 0.077 | 94.54 | 1227.79 | [S49] |
| 5 | MXene/AgNW/PVA/PCCs | 0.04 | 52.8 | 1320 | [S50] |
| 6 | PVA/MXene/PCC | 0.04 | 52.4 | 1310 | [S51] |
| 7 | MXene/FA-CNF | 0.12 | 63.8 | 531.67 | [S52] |
| 8 | Fe_3_O_4_/MXene/PVA | 0.075 | 40.00 | 533.33 | [S53] |
| 9 | PTP@MXene/SXL | 0.028 | 21.69 | 774.64 | [S54] |
| 10 | SA-MMT/MXene | 0.038 | 50.01 | 1316.05 | [S55] |
| 11 | h-MXene/ANF | 0.12 | 78.9 | 657.5 | [S56] |
| 12 | TOCNF/CS/MXene | 0.5238 | 66.6 | 127 | [S57] |
| 13 | PI-MXene-MWCNT | 0.108 | 66.8 | 618.5 | [S58] |
| 14 | CNTs/MXene/CNF | 0.038 | 36.6 | 963.2 | [S59] |
| 15 | CNT/PP | 0.035 | 31.2 | 891.4 | [S60] |
| 16 | CF/PC/Ni | 0.31 | 72.7 | 234.5 | [S61] |
| 17 | CF@Fe_3_O_4_/(BN/SR) | 0.74 | 37.67 | 50.9 | [S6] |
| 18 | MXene/wood | 0.38 | 32.7 | 86.1 | [S62] |
| 19 | MXene-WPU-Co/C | 0.12 | 42 | 350 | [S63] |
| 1 | **S-XM_0_**  **S-XM_5_**  **S-XM_10_**  **S-XM_15_**  **S-XM_20_**  **S-XM_25_** | **0.200**  **0.100**  **0.070**  **0.060**  **0.040**  **0.035** | **37**  **43**  **47**  **50**  **53**  **56** | **185**  **430**  **671.43**  **833.33**  **1325**  **1600** | **This work** |

**Table S3** Comparison of photothermal performance data

|  | Materials | Radiation intensity (mW/cm^2^) | Temperature (°C) | Time to stabilization (sec.) | Refs. |
| --- | --- | --- | --- | --- | --- |
| 1 | MXene/AgNW/TPU/PDMS | 50 | 43 | >25 | [S64] |
| 2 | PPUO@CuS_2_ | 50 | 52.2 | >50 | [S65] |
| 3 | MXene/AgNW/PDMS/Cotton | 50 | 62 | >60 | [S66] |
| **4** | MXene/TPU | 50 | 42 | >250 | [S67] |
| 5 | MXene/modified sawdust | 50 | 48 | >25 | [S68] |
| 6 | PVA/MXene/PCC | 76 | 47.1 | 18 | [S51] |
| 7 | C-GO/MXene/Cellulose | 50 | 43 | >100 | [S69] |
| 8 | S-rGO/LM | 100 | 47.5 | >20 | [S70] |
| 9 | PDA/Cellulose/MXene | 100 | 46 | 20 | [S71] |
| 10 | MXene/PI | 300 | 36 | 10 | [S72] |
| 11 | MXene@Ag/PA-CO/NY | 50 | 46.8 | >50 | [S73] |
| 12 | rGO-C/MXene-PVA-PEG | 100 | 71.5 | 68 | [S74] |
| 13 | CuS@PEI/PA-CF | 100 | 99 | 120 | [S75] |
| 14 | TPU/PDA/Ag/Fe_3_O_4_ | 50 | 66.9 | 80 | [S76] |
| 15 | PANI/Fe_3_O_4_/carbon cloth | 50 | 50.3 | >100 | [S77] |
| 16 | NS/PDA/MXene/SiO_2_/FOTS | 60 | 51.2 | >100 | [S78] |
| 17 | MXene/HA | 50 | 33.3 | >15 | [S79] |
| 18 | ANF/PPy | 100 | 53 | >10 | [S80] |
| 19 | **S-XM_20_** | **45.51**  **69.37**  **87.51**  **117.42**  **141.61** | **56.38**  **67.89**  **78.33**  **90.14**  **105.52** | **<10** | **This work** |

**Table S4** Comparison of Joule heating performance data

|  | Materials | Applied voltage (V) | Temperature (°C) | Refs. |
| --- | --- | --- | --- | --- |
| 1 | CWF/ZIF-8 | 1  2  3  4 | 35  40  50  70 | [S81] |
| 2 | MXene/ANF | 0.5  1  1.5  2  2.5  3  3.5  4 | 23.5  28  34.6  47.6  70.1  94.9  122.2  146.1 | [S36] |
| 3 | PVA/MXene/PCC | 1  1.5  2  2.5  3 | 20.5  35.6  51.7  84.1  114.9 | [S51] |
| **4** | PPy/MXene/PET | 1  2  3  4 | 30  40  57  79 | [S32] |
| 5 | Si-TM/BC | 1  2  3  4 | 35  50  90  130 | [S82] |
| 6 | **S-XM_20_** | **1**  **1.5**  **2**  **2.5**  **3**  **3.5** | **39.6**  **51.8**  **76.9**  **110.9**  **141.1**  **201.3** | **This work** |

**Supplementary References**

1. P. Das, A. Katheria, J. Nayak, S. Das, K. Nath et al., Facile preparation of self-healable and recyclable multilayered graphene-based nanocomposites for electromagnetic interference shielding applications. Colloids Surf. A Physicochem. Eng. Asp. **676**, 132244 (2023). <https://doi.org/10.1016/j.colsurfa.2023.132244>
2. Z. Wei, Y. Cai, Y. Zhan, Y. Meng, N. Pan et al., Ultra‐low loading of ultra‐small Fe_3_O_4_ nanoparticles on nonmodified CNTs to improve green EMI shielding capability of rubber composites. Small **20**, 2307148 (2023). <https://doi.org/10.1002/smll.202307148>
3. Rahaman, M., Superior mechanical, electrical, dielectric, and EMI shielding properties of ethylene propylene diene monomer (EPDM) based carbon black composites. RSC Adv. **13**, 25443-25458 (2023). <https://doi.org/10.1039/d3ra04187e>
4. D. Yang, J. Tao, Y. Yang, Q. He, Y. Weng et al., Effect interfacial size and multiple interface on electromagnetic shielding of silicon rubber/carbon nanotube composites with mixing segregated particles. Compos. Struct. **292**, 115668 (2022). <https://doi.org/10.1016/j.compstruct.2022.115668>
5. J. Luo, S. Zhao, H. Zhang, Z. Deng, L. Li et al., Flexible, stretchable and electrically conductive MXene/natural rubber nanocomposite films for efficient electromagnetic interference shielding. Compos. Sci. Technol. **182**, 107754 (2019). <https://doi.org/10.1016/j.compscitech.2019.107754>
6. Y. Guo, H. Qiu, K. Ruan, S. Wang, Y. Zhang et al., Flexible and insulating silicone rubber composites with sandwich structure for thermal management and electromagnetic interference shielding. Compos. Sci. Technol. **219**, 109253 (2022). <https://doi.org/10.1016/j.compscitech.2021.109253>
7. X. Li, P. Zhao, L. Han, C. Deng, Hierarchically structured elastomer for absorption-dominated electromagnetic interference shielding in an ultra-wide band. Compos. Sci. Technol. **219**, 109221 (2022). <https://doi.org/10.1016/j.compscitech.2021.109221>
8. H. Li, X. Ru, Y. Song, H. Wang, C. Yang et al., Flexible sandwich-structured silicone rubber/MXene/Fe_3_O_4_ composites for tunable electromagnetic interference shielding. Ind. Eng. Chem. Res. **61**, 11766-11776 (2022). <https://doi.org/10.1021/acs.iecr.2c01419>
9. T. Chen, J. Cai, D. Gong, X. Cheng, P. Liu et al., Magnetically driven hierarchically ordered carbonyl iron@SiO_2_/Ni@Ag/silicone rubber composite film for enhanced electromagnetic interference shielding with ultralow reflection. J. Mater. Chem. C **11**, 6597-6606 (2023). <https://doi.org/10.1039/D3TC00626C>
10. S. Choudhury, P. Das, P. Bhawal, A. Pal, P. Banerji et al., Double percolation behavior through the preferential distribution of conductive black in polymer blends to boost electrical properties and EMI shielding effectiveness. Mater. Today Commun. **35**, 106109 (2023). <https://doi.org/10.1016/j.mtcomm.2023.106109>
11. Y. Zhan, E. Lago, C. Santillo, A. Del Río Castillo, S. Hao et al., An anisotropic layer-by-layer carbon nanotube/boron nitride/rubber composite and its application in electromagnetic shielding. Nanoscale 12, 7782-7791 (2020). <https://doi.org/10.1039/C9NR10672C>
12. W. Yang, J. Liu, L. Wang, W. Wang, A. Yuen et al., Multifunctional MXene/natural rubber composite films with exceptional flexibility and durability. Compos. B Eng. **188**, 107875 (2020). <https://doi.org/10.1016/j.compositesb.2020.107875>
13. L. Ma, W. Yang, C. Jiang, Stretchable conductors of multi-walled carbon nanotubes (MWCNTs) filled thermoplastic vulcanizate (TPV) composites with enhanced electromagnetic interference shielding performance. Compos. Sci. Technol. **195**, 108195 (2020). <https://doi.org/10.1016/j.compscitech.2020.108195>
14. S. Ran, J. Xie, C. Li, H. Qin, Z. Chen et al., Polydopamine-assisted silver-coated spherical boron nitride as dual functional filler for thermal management and electromagnetic interference shielding. Diamond Relat. Mater. **135**, 109856 (2023). <https://doi.org/10.1016/j.diamond.2023.109856>
15. Q. Song, B. Chen, Z. Zhou, C. Lu, Flexible, stretchable and magnetic Fe_3_O_4_@Ti_3_C_2_T_x_/elastomer with supramolecular interfacial crosslinking for enhancing mechanical and electromagnetic interference shielding performance. Sci. China Mater. **64**, 1437-1448 (2021). <https://doi.org/10.1007/s40843-020-1539-2>
16. X. Xu, X. Zhao, N. Vokhidova, Q. Sun, Y. Li et al., Aircraft coating for electromagnetic interference shielding with flame retardancy and Joule heating capability. Mater. Chem. Phys. **299**, 127539 (2023). <https://doi.org/10.1016/j.matchemphys.2023.127539>
17. P. Das, A. Katheria, S. Ghosh, B. Roy, J. Nayak et al., Self-healable and super-stretchable conductive elastomeric nanocomposites for efficient thermal management characteristics and electromagnetic interference shielding. Synth. Met. **294**, 117304 (2023). <https://doi.org/10.1016/j.synthmet.2023.117304>
18. X. Jiang, J. Zhou, X. Zhong, Z. Hu, R. Hu et al., Stretchable PEDOT:PSS/Li-TFSI/XSB composite films for electromagnetic interference shielding. ACS Appl. Mater. Interfaces **15**, 8521-8529 (2023). <https://doi.org/10.1021/acsami.2c21604>
19. Y. Wang, R. Liu, J. Zhang, M. Miao, X. Fen, Vulcanization of Ti_3_C_2_T MXene/natural rubber composite films for enhanced electromagnetic interference shielding. Appl. Surf. Sci. **546**, 149143 (2021). <https://doi.org/10.1016/j.apsusc.2021.149143>
20. C. Jiao, Z. Deng, P. Min, J. Lai, Q. Gou et al., Photothermal healable, stretchable, and conductive MXene composite films for efficient electromagnetic interference shielding. Carbon **198**, 179-187 (2022). <https://doi.org/10.1016/j.carbon.2022.07.017>
21. Y. Zhan, J. Wang, K. Zhang, Y. Li, Y. Meng et al., Fabrication of a flexible electromagnetic interference shielding Fe_3_O_4_@reduced graphene oxide/natural rubber composite with segregated network. Chem. Eng. J. **344**, 184-193 (2018). <https://doi.org/10.1016/j.cej.2018.03.085>
22. S. Ghosh, S. Remanan, S. Mondal, S. Ganguly, P. Das et al., An approach to prepare mechanically robust full IPN strengthened conductive cotton fabric for high strain tolerant electromagnetic interference shielding. Chem. Eng. J. **344**, 138-154 (2018). <https://doi.org/10.1016/j.cej.2018.03.039>
23. C. Liu, J. Cai, P. Dang, X. Li, D. Zhang, Highly stretchable electromagnetic interference shielding materials made with conductive microcoils confined to a honeycomb structure. ACS Appl. Mater. Interfaces **12**, 12101-12108 (2020). <https://dx.doi.org/10.1021/acsami.0c00034>
24. G. Wang, X. Liao, J. Yang, W. Tang, Y. Zhang et al., Frequency-selective and tunable electromagnetic shielding effectiveness via the sandwich structure of silicone rubber/graphene composite. Composites Science and Technology **184**, 107847 (2019). <https://doi.org/10.1016/j.compscitech.2019.107847>
25. Z. Sun, Y. Dong, W. Zhang, Y. Liu, X. Tian et al., Liquid metal/elastomer composites with strain invariant electromagnetic shielding. Compos. Commun. **56**, 102364 (2025). <https://doi.org/10.1016/j.coco.2025.102364>
26. Y. Yang, L. Shao, J. Wang, W. Wang, C. Su et al., Flexible and multifunctional carboxylic styrene butadiene rubber/CNT@PDA/MXene composites film for effective electromagnetic interference shielding, thermal management, and energy harvesting. Compos. Commun. **54**, 102267 (2025). <https://doi.org/10.1016/j.coco.2025.102267>
27. T. Chen, X. Cheng, J. Cai, D. Gong, W. Gong et al., Robust multifunctional films with excellent EMI shielding, anti-peeling, and Joule heating performances enabled by an encapsulated highly conductive fabric strategy. Small **21**, 2409033 (2025). <https://doi.org/10.1002/smll.202409033>
28. Z. Ye, D. Zhao, F. Liu, J. Luo, X. Liu et al., Flexible and highly conductive Ti_3_C_2_T_x_/natural rubber composites with interconnected networks for high-performance electromagnetic interference shielding. Compos. A Appl. Sci. Manuf. **180**, 108067 (2024). <https://doi.org/10.1016/j.compositesa.2024.108067>
29. X. Jiang, Z. Hu, S. Xu, Y. Song, Q. Zheng, PEDOT:PSS/XNBR films for low-temperature stretchable electromagnetic interference shielding. Compos. Commun. **50**, 102029 (2024). <https://doi.org/10.1016/j.coco.2024.102029>
30. Z. Wei, Y. Cai, Y. Zhan, Y. Meng, N. Pan et al., Ultra-low loading of ultra-small Fe_3_O_4_ nanoparticles on nonmodified CNTs to improve green EMI shielding capability of rubber composites. Small **20**, 2307148 (2024). <https://doi.org/10.1002/smll.202307148>
31. Z. Yu, C. Deng, J. Sun, X. Zhang, Y. Liu et al., Cellulosic nonwovens incorporated with fully utilized MXene precursor as smart pressure sensor and multi‐protection materials. Adv. Funct. Mater. **34**, 2402707 (2024). <https://doi.org/10.1002/adfm.202402707>
32. Q. Wang, H. Zhang, J. Liu, S. Zhao, X. Xie et al., Multifunctional and water‐resistant MXene‐decorated polyester textiles with outstanding electromagnetic interference shielding and Joule heating performances. Adv. Funct. Mater. **29**, 1806819 (2018). <https://doi.org/10.1002/adfm.201806819>
33. P. Yi, H. Zou, Y. Yu, X. Li, Z. Li et al., MXene-reinforced liquid metal/polymer fibers via interface engineering for wearable multifunctional textiles. ACS Nano **16**, 14490-14502 (2022). <https://doi.org/10.1021/acsnano.2c04863>
34. X. Zheng, W. Cao, X. Hong, L. Zou, Z. Liu et al., Versatile electronic textile enabled by a mixed‐dimensional assembly strategy. Small **19**, 2208134 (2023). <https://doi.org/10.1002/smll.202208134>
35. M. Cheng, M. Ying, R. Zhao, L. Ji, H. Li et al., Transparent and flexible electromagnetic interference shielding materials by constructing sandwich AgNW@MXene/wood composites. ACS Nano **16**, 16996-17007 (2022). <https://doi.org/10.1021/acsnano.2c07111>
36. J. Wang, X. Ma, J. Zhou, F. Du, C. Teng, Bioinspired, high-strength, and flexible MXene/aramid fiber for electromagnetic interference shielding papers with Joule heating performance. ACS Nano **16**, 6700-6711 (2022). <https://doi.org/10.1021/acsnano.2c01323>
37. Y. Zhang, K. Ruan, K. Zhou, J. Gu, Controlled distributed Ti_3_C_2_T_x_ hollow microspheres on thermally conductive polyimide composite films for excellent electromagnetic interference shielding. Adv. Mater. **35**, 2211642 (2023). <https://doi.org/10.1002/adma.202211642>
38. Z. Xie, L. Yao, H. Fang, Z. Yang, X. Zhou et al., Multi‐functional and flexible nano‐silver@MXene heterostructure‐decorated graphite felt for wearable thermal therapy. Small **20**, 2310191 (2024). <https://doi.org/10.1002/smll.202310191>
39. K. Liu, H. Du, W. Liu, M. Zhang, Y. Wang et al., Strong, flexible, and highly conductive cellulose nanofibril/PEDOT:PSS/MXene nanocomposite films for efficient electromagnetic interference shielding. Nanoscale **14**, 14902-14912 (2022). <https://doi.org/10.1039/D2NR00468B>
40. Y. Li, Y. Wang, Z. Li, S. Ma, Y. Zhang et al., Meter-scale wearable multifunctional core-shell nanofiber textiles for ultra-broadband electromagnetic interference shielding and infrared stealth. Adv. Mater. **37**, 2501485 (2025). <https://doi.org/10.1002/adma.202501485>
41. M. Song, Z. Liu, Y. Wang, C. Liu, J. Guo et al., Industrial-grade flexible carbon fiber paper/MXene composite electromagnetic shielding material with ultra-large area and ultra-high performance. Adv. Funct. Mater. **35**, 2421422 (2025). <https://doi.org/10.1002/adfm.202421422>
42. M. Liu, H. Zhang, X. Huang, Z. Zhang, K. Zhang et al., An electric-magnetic dual-gradient composite film comprising MXene, hollow Fe_3_O_4_, and bacterial cellulose for high-performance EMI shielding and infrared camouflage. Adv. Funct. Mater. **35**, 2419077 (2025). <https://doi.org/10.1002/adfm.202419077>
43. M. Ma, X. Liao, Q. Chu, S. Chen, Y. Shi et al., Construction of gradient conductivity cellulose nanofiber/MXene composites with efficient electromagnetic interference shielding and excellent mechanical properties. Compos. Sci. Technol. **226**, 109540 (2022). <https://doi.org/10.1016/j.compscitech.2022.109540>
44. H. Liu, Z. Cui, L. Luo, Q. Liao, R. Xiong et al., Facile fabrication of flexible and ultrathin self-assembled Ti_3_C_2_T_x_/bacterial cellulose composite films with multifunctional electromagnetic shielding and photothermal conversion performances. Chem. Eng. J. **454**, 140288 (2023). <https://doi.org/10.1016/j.cej.2022.140288>
45. Z. Zhan, Q. Song, Z. Zhou, C. Lu, Ultrastrong and conductive MXene/cellulose nanofiber films enhanced by hierarchical nano-architecture and interfacial interaction for flexible electromagnetic interference shielding. J. Mater. Chem. C **7**, 9820-9829 (2019). <https://doi.org/10.1039/C9TC03309B>
46. L. Li, Z. Zhao, Y. Pan, M. Chen, Y. Zhang et al., In-situ growth of CuS on polyimide film to construct dense and continuous network: Achieving excellent electrothermal and EMI shielding performance. Compos. Sci. Technol. **250**, 110543 (2024). <https://doi.org/10.1016/j.compscitech.2024.110543>
47. Y. Zhang, Q. Gao, X. Sheng, S. Zhang, J. Chen et al., Flexible, robust, sandwich structure polyimide composite film with alternative MXene and Ag NWs layers for electromagnetic interference shielding. J. Mater. Sci. Technol. **159**, 194-203 (2023). <https://doi.org/10.1016/j.jmst.2022.10.091>
48. Y. Duan, P. Xu, T. Liu, Z. Wang, X. Yan et al., Design of PDMS/SiO_2_@MXene composites with “Floatable Interlayer” structure for the electromagnetic shielding behavior improvement. Chem. Eng. J. **461**, 141853 (2023). <https://doi.org/10.1016/j.cej.2023.141853>
49. G. Hu, F. Dong, J. Xu, Y. Xiong, High thermally conductive, hydrophobic and small-thickness nanocomposite films with symmetrical double conductive networks exhibit ultra-high electromagnetic shielding performance. Compos. A Appl. Sci. Manuf. **167**, 107416 (2023). <https://doi.org/10.1016/j.compositesa.2022.107416>
50. Y. Ding, X. Lu, S. Liu, H. Wu, X. Sheng et al., Sandwich-structured multifunctional composite films with excellent electromagnetic interference shielding and light/electro/magnetic-to-thermal conversion and storage capabilities. Compos. A Appl. Sci. Manuf. **163**, 107168 (2022). <https://doi.org/10.1016/j.compositesa.2022.107178>
51. S. Gong, X. Sheng, X. Li, M. Sheng, H. Wu et al., A multifunctional flexible composite film with excellent multi‐source driven thermal management, electromagnetic interference shielding, and fire safety performance, inspired by a “brick-mortar” sandwich structure. Adv. Funct. Mater. **32**, 2200570 (2022). <https://doi.org/10.1002/adfm.202200570>
52. Z. Guo, P. Ren, J. Wang, J. Tang, F. Zhang et al., Multifunctional sandwich-structured magnetic-electric composite films with Joule heating capacities toward absorption-dominant electromagnetic interference shielding. Compos. B Eng. **236**, 109836 (2022). <https://doi.org/10.1016/j.compositesb.2022.109836>
53. Y. Zhang, K. Ruan, J. Gu, Flexible sandwich‐structured electromagnetic interference shielding nanocomposite films with excellent thermal conductivities. Small **17**, 2101951 (2021). <https://doi.org/10.1002/smll.202101951>
54. Y. Wang, H. Peng, T. Li, B. Shiu, H. Ren et al., MXene-coated conductive composite film with ultrathin, flexible, self-cleaning for high-performance electromagnetic interference shielding. Chem. Eng. J. **412**, 128681 (2021). [https://doi.org/10.1016/j.cej.2021.128681](https://doi.org/10.1016/j.cej.2021.128681)
55. Y. Zhang, W. Cheng, W. Tian, J. Lu, L. Song et al., Nacre-inspired tunable electromagnetic interference shielding sandwich films with superior mechanical and fire-resistant protective performance. ACS Appl. Mater. Interfaces **12**, 6371-6382 (2020). https://dx.doi.org/10.1021/acsami.9b18750
56. F. Xie, Q. Liu, L. Zhuo, H. Wei, Y. Shang et al., Hierarchically engineered sandwich-structured h-MXene/ANF hybrid films with tunable electromagnetic interference shielding and exceptional environmental resilience. Compos. Struct. **367**, 119248 (2025). <https://doi.org/10.1016/j.compstruct.2025.119248>
57. J. Wei, P. He, Y. Yang, C. Tang, Z. Long et al., Enhancing the electromagnetic interference shielding performance of composite paper with corrugated paper-like structure. Chem. Eng. J. **497**, 154969 (2024). <https://doi.org/10.1016/j.cej.2024.154969>
58. W. Liang, J. Wu, S. Zhang, P. Zhao, X. Zuo et al., Construction of PI-MXene-MWCNT nanocomposite film integrating conductive gradient with sandwich structure for high-efficiency electromagnetic interference shielding in extreme environments. Carbon **228**, 119328 (2024). <https://doi.org/10.1016/j.carbon.2024.119328>
59. W. Cao, C. Ma, S. Tan, M. Ma, P. Wan et al., Ultrathin and flexible CNTs/MXene/cellulose nanofibrils composite paper for electromagnetic interference shielding. Nano-Micro Lett. **11**, 72 (2019). <https://doi.org/10.1007/s40820-019-0304-y>
60. Y. Hu, D. Li, L. Wu, J. Yang, X. Jian et al., Carbon nanotube buckypaper and buckypaper/polypropylene composites for high shielding effectiveness and absorption-dominated shielding material. Compos. Sci. Technol. **181**, 107699 (2019). <https://doi.org/10.1016/j.compscitech.2019.107699>
61. D. Xing, L. Lu, K. Teh, Z. Wan, Y. Xie et al., Highly flexible and ultra-thin Ni-plated carbon-fabric/polycarbonate film for enhanced electromagnetic interference shielding. Carbon **132**, 32-41 (2018). [https://doi.org/10.1016/j.carbon.2018.02.001](https://doi.org/10.1016/j.carbon.2018.02.001)
62. Y. Jiang, X. Ru, W. Che, Z. Jiang, H. Chen et al., Flexible, mechanically robust and self-extinguishing MXene/wood composite for efficient electromagnetic interference shielding. Compos. B Eng. **229**, 109460 (2022). <https://doi.org/10.1016/j.compositesb.2021.109460>
63. L. Ran, L. Qiu, H. Zhao, F. Sun, Z. Chen et al., Fabrication of MXene based sandwich-like films for excellent flexibility, electromagnetic interference shielding and thermal management. Compos. A Appl. Sci. Manuf. **173**, 107672 (2023). <https://doi.org/10.1016/j.compositesa.2023.107672>
64. J. Dong, Y. Feng, K. Lin, B. Zhou, F. Su et al., A stretchable electromagnetic interference shielding fabric with dual‐mode passive personal thermal management. Adv. Funct. Mater. **34**, 2310774 (2023). <https://doi.org/10.1002/adfm.202310774>
65. J. Wu, C. Zhu, H. Morikawa, X. Zhang, X. Yin et al., A breathable fibrous membrane with coaxially heterogeneous conductive networks toward personal thermal management and electromagnetic interference shielding. Small **20**, 2311827 (2024). <https://doi.org/10.1002/smll.202311827>
66. L. Tang, B. Lyu, D. Gao, Z. Jia, Y. Fu et al., A Janus textile with tunable heating modes toward precise personal thermal management in cold conditions. Small **20**, 2308194 (2023). <https://doi.org/10.1002/smll.202308194>
67. M. Feng, S. Feng, T. Yu, S. Zhu, H. Cai et al., Versatile and comfortable Janus fabrics for switchable personal thermal management and electromagnetic interference shielding. Adv. Fiber Mater. **6**, 911-924 (2024). <https://doi.org/10.1007/s42765-024-00393-w>
68. P. Wang, T. Mai, W. Zhang, M. Qi, L. Chen et al., Robust and multifunctional Ti_3_C_2_T_x_/modified sawdust composite paper for electromagnetic interference shielding and wearable thermal management. Small **20**, 2304914 (2023). <https://doi.org/10.1002/smll.202304914>
69. B. Li, N. Wu, Y. Yang, F. Pan, C. Wang et al., Graphene oxide‐assisted multiple cross‐linking of MXene for large‐area, high‐strength, oxidation‐resistant, and multifunctional films. Adv. Funct. Mater. **33**, 2213357 (2022). <https://doi.org/10.1002/adfm.202213357>
70. Y. Sun, X. Han, P. Guo, Z. Chai, J. Yue et al., Slippery graphene-bridging liquid metal layered heterostructure nanocomposite for stable high-performance electromagnetic interference shielding. ACS Nano **17**, 12616-12628 (2023). <https://doi.org/10.1021/acsnano.3c02975>
71. Q. Liu, P. Wang, W. Zhang, T. Mai, M. Qi et al., Multifunctional wood-derived cellulose/Ti_3_C_2_T_x_ composite films enhanced by densification strategy for electromagnetic shielding, Joule/solar heating, and thermal camouflage. Chem. Eng. J. **493**, 152696 (2024). <https://doi.org/10.1016/j.cej.2024.152696>
72. J. Chen, X. Hu, Z. Nie, Y. Feng, S. Qi et al., Flexible multilayer MXene/Polyimide composite film with excellent electromagnetic interference shielding and photothermal conversion performance. J. Alloys Compounds **990**, 174399 (2024). <https://doi.org/10.1016/j.jallcom.2024.174399>
73. J. Liu, J. Zhang, X. Cui, W. Gu, Q. Liu et al., Eco-friendly wearable textiles: Asymmetric structures for EMI shielding, thermal management, and fire safety. Chem. Eng. J. **491**, 152097 (2024). <https://doi.org/10.1016/j.cej.2024.152097>
74. B. Hu, H. Guo, T. Li, J. Li, M. Cao et al., Millefeuille-like phase change composite with multi-level oriented architecture for efficient thermal management and electromagnetic interference shielding. Chem. Eng. J. **483**, 149240 (2024). <https://doi.org/10.1016/j.cej.2024.149240>
75. Z. Zhao, X. Song, Q. Zhang, Y. Zhang, Q. Feng et al., Copper sulfide and polyelectrolyte decorated textiles for active/passive personal thermal management. Chem. Eng. J. **480**, 148258 (2024). <https://doi.org/10.1016/j.cej.2023.148258>
76. Y. Zhang, X. Tang, H. Li, Z. Zhao, Y. Pan et al., Bilayered distribution of Ag and Fe_3_O_4_ in electrospun TPU films for low-reflection electromagnetic interference shielding and multiple thermal management functionalities. Ind. Eng. Chem. Res. **63**, 318-329 (2023). <https://doi.org/10.1021/acs.iecr.3c03723>
77. L. Wang, M. Zhou, H. Fu, An in-situ growth Fe_3_O_4_ and polyaniline on carbon cloth encapsulated composite phase change materials with high thermal conductivity and photothermal energy conversion and storage. J. Energy Storage **78**, 110090 (2024). <https://doi.org/10.1016/j.est.2023.110090>
78. J. Peng, H. Cheng, J. Liu, W. Han, T. Wu et al., Superhydrophobic MXene-based fabric with electromagnetic interference shielding and thermal management ability for flexible sensors. Adv. Fiber Mater. **5**, 2099-2113 (2023). <https://doi.org/10.1007/s42765-023-00328-x>
79. J. Xiong, R. Ding, Z. Liu, H. Zheng, P. Li et al., High-strength, super-tough, and durable nacre-inspired MXene/heterocyclic aramid nanocomposite films for electromagnetic interference shielding and thermal management. Chem. Eng. J. **474**, 145972 (2023). <https://doi.org/10.1016/j.cej.2023.145972>
80. D. Guo, C. Mu, Q. Liu, B. Wang, J. Xiang et al., Aramid nanofiber/polypyrrole composite films for broadband EMI shielding, wearable electronics, Joule heating, and photothermal conversion. ACS Appl. Nano Mater. **6**, 15108-15118 (2023). <https://doi.org/10.1021/acsanm.3c02645>
81. X. Ma, J. Pan, H. Guo, J. Wang, C. Zhang et al., Ultrathin wood‐derived conductive carbon composite film for electromagnetic shielding and electric heating management. Adv. Funct. Mater. **33**, 2213431 (2023). <https://doi.org/10.1002/adfm.202213431>
82. Z. Zhou, Q. Song, B. Huang, S. Feng, C. Lu, Facile fabrication of densely packed Ti_3_C_2_ MXene/nanocellulose composite films for enhancing electromagnetic interference shielding and electro-/photothermal performance. ACS Nano **15**, 12405-12417 (2021). <https://doi.org/10.1021/acsnano.1c04526>
